# Cell‐based polygenic risk scores predict clinical progression and prioritize network‐based therapeutic targets in Alzheimer's disease

**DOI:** 10.1002/alz.71895

**Published:** 2026-10-06

**Authors:** Nathan Sahelijo, Priya Rajagopalan, Lu Qian, Rufuto Rahman, Daniel Goldstein, Sophia I. Thomopoulos, David A. Bennett, Lindsay A. Farrer, Thor D. Stein, Li Shen, Heng Huang, Kwangsik Nho, Andrew J. Saykin, Christos Davatzikos, Paul M. Thompson, Julia TCW, Gyungah R. Jun

**Affiliations:** ^1^ Biomedical Genetics Section Department of Medicine Boston University Chobanian and Avedisian School of Medicine Boston Massachusetts USA; ^2^ Bioinformatics Program Faculty of Computing & Data Sciences Boston University Boston Massachusetts USA; ^3^ Imaging Genetics Center Mark & Mary Stevens Neuroimaging & Informatics Institute Keck School of Medicine University of Southern California Los Angeles California USA; ^4^ Department of Neurology Keck School of Medicine University of Southern California Los Angeles California USA; ^5^ Department of Pharmacology Physiology & Biophysics Boston University Chobanian and Avedisian School of Medicine Boston Massachusetts USA; ^6^ Rush Alzheimer's Disease Center Rush University Medical Center Chicago Illinois USA; ^7^ Boston University Alzheimer's Disease Research Center Boston University Chobanian and Avedisian School of Medicine Boston Massachusetts USA; ^8^ Department of Biostatistics Boston University School of Public Health Boston Massachusetts USA; ^9^ Department of Epidemiology Boston University School of Public Health Boston Massachusetts USA; ^10^ Department of Neurology Boston University Chobanian and Avedisian School of Medicine Boston Massachusetts USA; ^11^ Department of Pathology & Laboratory Medicine Boston University School of Medicine Boston Massachusetts USA; ^12^ VA Bedford Healthcare System Bedford Massachusetts USA; ^13^ VA Boston Healthcare Center Boston Massachusetts USA; ^14^ Departments of Biostatistics, Epidemiology, and Informatics University of Pennsylvania School of Medicine Philadelphia Pennsylvania USA; ^15^ Department of Computer Science University of Maryland College Park College Park Maryland USA; ^16^ Department of Radiology and Imaging Sciences and Medical and Molecular Genetics Indiana University School of Medicine Indianapolis Indiana USA; ^17^ Indiana Alzheimer's Disease Research Center Indiana University School of Medicine Indianapolis Indiana USA; ^18^ Center for Computational Biology and Bioinformatics Indiana University School of Medicine Indianapolis Indiana USA; ^19^ Department of Radiology University of Pennsylvania School of Medicine Philadelphia Pennsylvania USA

**Keywords:** Alzheimer's disease, cell‐based polygenic risk score, cell‐level co‐expression network, cognitive performance, drug repurposing, PageRank algorithm, patient stratification, precision medicine

## Abstract

**BACKGROUND:**

Late‐onset Alzheimer's disease (AD) exhibits substantial biological heterogeneity. We developed a framework linking cell‐type–specific polygenic risk profiles to precision medicine in AD.

**METHODS:**

Cell‐based polygenic risk scores (cbPRSs) derived from single‐nucleus RNA‐seq co‐expression networks were evaluated in Alzheimer's Disease Neuroimaging Initiative (ADNI) and Framingham Heart Study (FHS) cohorts. Network hubs were prioritized using a graph‐based PageRank algorithm and candidate drugs were validated in human‐induced pluripotent stem cell (hiPSC)–derived astrocytes.

**RESULTS:**

Cell‐based PRS analysis identified two genetic risk axes independent of network cell‐type labels: an apolipoprotein E (*APOE*)–concentrated axis (Ast‐M2/Oli‐M45) and an *APOE*‐independent axis (Ast‐M10/Oli‐M50), which predicted accelerated progression to AD (hazard ratio: 1.25–2.02) and correlated with localized temporal lobe atrophy, reduced glucose metabolism, and global amyloid burden. High‐risk status specifically upregulated complement C4a protein expression in postmortem brains. PageRank network analysis identified four candidate drugs targeting the *APOE*‐containing astrocyte network. Experimental treatment with estradiol and levetiracetam significantly reduced *APOE* and complement *C4* gene expression in hiPSC‐derived astrocytes.

**DISCUSSION:**

By integrating cell‐based genetic risk with network‐level target prioritization, this framework enables robust patient stratification and experimental target validation.

## Highlights

- Developed cell‐based polygenic risk scores (cbPRSs) to profile genetic risk within precise astrocyte and oligodendrocyte networks.
- High‐risk status in these networks predicts faster clinical progression to Alzheimer's disease (AD) in Framingham Heart Study (FHS) and Alzheimer's Disease Neuroimaging Initiative (ADNI) cohorts.
- High cbPRS correlates with reduced glucose metabolism and localized gray matter atrophy in the temporal lobe.
- Network priority hubs linked genetic risk profiles directly to increased *C4A* expression and neuroinflammation in astrocytes and oligodendrocytes.
- Estradiol and levetiracetam reversed apolipoprotein E (*APOE*), *C4A*, and *C4B* in human‐induced pluripotent stem cell (hiPSC)–derived astrocytes.

## 1 BACKGROUND

Late‐onset Alzheimer's disease (AD) is a progressive neurodegenerative disorder and the most common cause of dementia worldwide.^1^ Neuropathologically, AD is defined by extracellular amyloid beta (Aβ) plaques and intracellular tau neurofibrillary tangles, but individuals with AD show substantial variability in cognitive presentation, neuroanatomic involvement, and disease progression.^2^, ^3^, ^4^ This heterogeneity complicates the mechanistic interpretation of AD and suggests that multiple biological processes contribute to disease expression across individuals.

Genetic studies indicate that late‐onset AD is highly polygenic, with heritability estimates exceeding 50%.^5^ Genome‐wide association studies have identified dozens of risk loci implicating pathways related to lipid metabolism, immune function, and endosomal trafficking.^6^ However, these genetic findings have not yet translated into effective disease‐modifying therapies, likely reflecting the small effect sizes of individual variants and an incomplete understanding of their biological functions.^7^ Polygenic risk scores (PRSs), which aggregate the effect sizes of all risk alleles, provide an efficient measure of genetic liability.^8^ A recent study demonstrated that PRSs derived from co‐expressed networks in brain tissues explain a portion of clinical heterogeneity in patients with AD.^9^ Heterogeneous disease mechanisms may also drive this phenotypic variability; a previous study identified distinct patterns of cellular composition in the brains of individuals carrying AD‐associated genetic variants.^10^ These findings suggest that genetic risk for AD may influence cell‐type–specific processes in the brain. A cellular‐level perspective may inform more precise AD treatments.

We developed a framework for cell‐type–resolved genetic risk profiling in AD by integrating single‐nucleus RNA sequencing–derived gene networks with genomic and phenotypic data from two independent cohorts. We constructed cell‐based PRSs (cbPRSs) reflecting genetic risk within astrocyte‐ and oligodendrocyte‐specific networks and examined associations with cognitive decline, neuroimaging measures, and proteomic signatures. To assess the biological coherence of these network‐defined risk profiles, we evaluated whether prioritized network targets could be experimentally perturbed by existing drugs in human‐induced pluripotent stem cell (hiPSC)–derived astrocytes. This integrative approach links polygenic risk to cell‐type–specific molecular pathways and supports a framework for cell‐based precision medicine in AD.

**RESEARCH IN CONTEXT**

1. **Systematic review**: The authors reviewed the literature using public repositories, clinical trial databases, and recent publications on genome‐wide polygenic risk scores (gwPRSs) and cell‐type–specific disease mechanisms in late‐onset Alzheimer's disease (AD). Although gwPRSs efficiently aggregate overall genetic liability, they lack cellular resolution. Recent single‐nucleus RNA sequencing studies demonstrate that AD risk loci alter cerebral cortex cell‐type composition and network expression. However, effectively linking network‐level polygenic risk to distinct clinical phenotypes, neuroimaging endophenotypes, and targetable molecular hubs has remained an ongoing challenge in precision medicine.
2. **Interpretation**: This study presented an integrative framework that successfully resolved polygenic risk at the cellular level by constructing network‐specific cell‐based PRSs (cbPRSs) for astrocytes and oligodendrocytes. Evaluating these scores across the Alzheimer's Disease Neuroimaging Institute (ADNI) and Framingham Heart Study (FHS) cohorts demonstrated that cell‐based genetic risk profiles accurately predicted accelerated cognitive decline, localized temporal lobe gray matter loss, and elevated global amyloid burden. By using a graph‐based PageRank algorithm, the framework compressed massive co‐expression networks to prioritize key regulatory hubs, identifying apolipoprotein E (*APOE*) and the complement pathway (*C4A*) as primary drivers. Experimental validation in isogenic hiPSC‐derived astrocytes confirmed that candidate drugs (estradiol, levetiracetam, and pioglitazone) modulated expression of *APOE* and *C4A*.
3. **Future directions**: Future research should focus on validating these network‐driven cbPRSs in highly diverse, non‐European ancestry populations to ensure broader generalizability, as the current genome‐wide association study (GWAS) framework is based on European cohorts. In addition, we can extend to different subtypes from neuropathological and biomarker risk profiles. Finally, the candidate drugs identified must be tested in advanced multi‐cellular co‐culture models (integrating astrocytes, microglia, neurons, and oligodendrocytes) to fully capture cell–cell signaling dynamics and optimize patient stratification protocols for upcoming precision medicine clinical trials.

## 2 MATERIALS AND METHODS

### 2.1 Identifying brain cell‐level co‐expression networks

We obtained single nuclei RNA sequencing (snRNA‐seq) data from the CommonMind Consortium portal (http://www.synapse.org). The discovery set contained snRNA‐seq data from the dorsolateral prefrontal cortex (DLFPC) area of 48 autopsied brains with 24 AD cases and 24 controls in the Religious Orders Study and Rush Memory and Aging Project (ROSMAP). Clinical and neuropathologic evaluation procedures have been described previously.^11^, ^12^, ^13^, ^14^ Protocols were approved by the Rush University Medical Center Institutional Review Board (IRB). For validation, snRNA‐seq data from DLPFC tissue of 12 AD cases and 9 controls were obtained from the Southwest Dementia Brain Bank (SWDBB). Major brain cell classes were annotated using established marker genes. Read counts were aligned to GRCh38 using CellRanger (v.3.1.0); a 200‐UMI cutoff was used to retain low‐abundance populations. Gene expression was log‐normalized (library‐size scaled by 10,000 and log‐transformed) and adjusted for total counts. Dimensionality reduction using the top 10 principal components followed by t‐distributed stochastic neighbor embedding (t‐SNE) analysis, with cell‐type labels assigned by known marker genes, yielded eight cell‐type clusters.^15^ Pericytes were excluded due to their omission in the validation dataset. Cell‐type–specific gene co‐expression networks were constructed using weighted gene correlation network analysis (WGCNA),^16^ with individual nuclei rather than pseudobulk donor aggregates serving as observations. Prior to network construction, cells were filtered using cell‐type–specific nFeature thresholds (lower bound: 150 features; upper bound: 99th percentile per cell type), and genes with summed expression ≤10 across all cells were excluded. Signed co‐expression networks were constructed using topological overlap matrices (TOMs), with soft‐thresholding powers chosen per cell type (range: 7–17) to satisfy approximate scale‐free topology (R^2^ ≥ 0.80). Modules were identified by average‐linkage hierarchical clustering of the TOM dissimilarity matrix with dynamic tree cutting. Network preservation was evaluated in the SWDBB dataset, and preserved modules with Z‐summary ≥10 were retained for downstream analyses.^17^

### 2.2 Calculating cbPRSs in two independent studies

Cell‐based PRSs (cbPRSs) were generated in two independent cohorts, the Alzheimer's Disease Neuroimaging Initiative (ADNI) and the Framingham Heart Study (FHS). ADNI is a longitudinal, multicenter study tracking AD progression with clinical, imaging, genetic, and biomarker data. We used the combined ADNI 1‐3 dataset of 1414 participants,^18^ with genotypic and phenotypic data retrieved from the Laboratory of Neuro Imaging (LONI) website (http://adni.loni.usc.edu). The FHS provided a community‐based longitudinal cohort with ongoing dementia surveillance (*n* = 8481).^19^ Both studies followed Good Clinical Practice guidelines and IRB approval.

We computed cbPRSs using genome‐wide significant AD‐associated single nucleotide polymorphisms (SNPs; *p* < 5.4×10^−^^8^) from the Kunkle et al. (2019)^20^ European‐ancestry genome‐wide association study (GWAS) meta‐analysis for AD risk, located within 50 kb of genes belonging to a preserved cell‐type–specific co‐expression module. Variants were filtered for minor allele frequency (MAF) ≥1% and imputation quality R^2^ ≥ 0.3. Linkage Disequilibrium (LD) based clumping was performed using PLINK with the 1000 Genomes Project Phase 1 European reference panel (hg19), with parameters r^2^ ≤ 0.5 and a 250 kb window; strand‐ambiguous SNPs were excluded prior to clumping. Each cbPRS was calculated as the weighted sum of Haplotype Reference Consortium (HRC)‐imputed risk allele dosages, with weights derived from Kunkle 2019 effect size estimates.^20^ Apolipoprotein E (*APOE*)‐region variants were included when *APOE* was a member of the module (Ast‐M2 and Oli‐M45). A genome‐wide PRS was also computed for comparison including *APOE*‐region variants. We calculated cbPRSs separately for ADNI and FHS datasets and across ADNI phases (ADNI1, ADNI2/GO, ADNI3, combined ADNI1‐3).

### 2.3 Association between cbPRSs and previously defined subtypes of AD in ADNI

We compared cbPRS distributions between cognitively normal (CN), mild cognitive impairment (MCI), and AD groups using *t*‐tests, and modeled cbPRS–AD diagnosis associations with logistic regression adjusted for age at last exam and sex. In the ADNI 1‐2/GO subset, age‐, and sex‐adjusted logistic regression assessed whether cbPRS predicted membership in previously defined cognitive subtypes^21^, ^22^, ^23^, ^24^ and neuroimaging atrophy clusters.^2^, ^4^ The cognitive subtypes consisted of AD‐executive function (*n* = 16), AD‐language (*n* = 48), AD‐memory (*n* = 184), AD‐multi‐domain (*n* = 25), AD‐no‐domain (275), and AD‐visuospatial (*n* = 85) subgroups. Atrophy clusters were defined by distinct patterns in AD and CN individuals (four clusters total). Each subject was assigned to a single cognitive subtype or atrophy cluster (coded as 1), with no overlap at last exam. Multiple‐testing correction was applied using a Bonferroni threshold (*p* < 1.67 × 10^−3^).

### 2.4 Cell‐based risk status association with AD phenotypes

Participants were classified as high risk (1) or low risk (0) for each cbPRS using quartile (first vs fourth) and median thresholds from the cbPRS distribution (cell‐based risk status). This stratification was applied in downstream phenotype analyses.

#### 2.4.1 Endophenotypes in ADNI

Endophenotypes in ADNI included global amyloid accumulation and temporal glucose uptake from positron emission tomography (PET) scans, hippocampal volume and entorhinal cortex thickness from magnetic resonance imaging (MRI) scans, and executive and memory composite scores. Endophenotypes were rank‐transformed after adjusting for age and sex. Associations were evaluated by logistic regression with binary risk status as outcome and additional adjustment for intracranial volume on MRI measures. Stratified analyses by last‐exam diagnosis (CN, MCI, AD) assessed disease‐stage‐dependent effects.

#### 2.4.2 Quantitative voxel‐based volumes in ADNI

Voxel‐based morphometry (VBM) using SPM12 was applied to segment T1‐weight MRI scans as part of the ENIGMA‐VBM initiative. Voxel‐wise gray matter, white matter, and cerebrospinal fluid (CSF) volumes were derived via deformation mapping against a priori tissue probability map (TPM). Analyses used MATLAB and Statistical Parametric Mapping (SPM) 12 (http://www.fil.ion.ucl.ac.uk/spm) within the Enhancing Neuro Imaging Genetics Through Meta Analysis consortium's voxel based morphometry (ENIGMA‐VBM) pipeline (https://sites.google.com/view/enigmavbm).^25^ MRI scans from 677 ADNI 1‐3 participants were preexisting, de‐identified, three‐dimensional sagittal magnetization prepared rapid gradient echo (MPRAGE) T1‐weighted images acquired on 1.5T or 3T scanners. Voxel‐wise differential gray‐matter volumes were modeled with binary risk status as outcome, controlling for age, sex, intracranial volume (ICV), and scanner field strength. False discovery rate (FDR) correction was applied across all gray‐matter voxels at *q* = 0.05, and the corresponding threshold *p*‐value is reported.

#### 2.4.3 Differential progression rate to AD in FHS and ADNI

Differential progression rate from CN or MCI to AD during the follow‐up examinations for cell‐based risk status was evaluated using Cox proportional hazards regression models separately in FHS and ADNI. Models included age interval between baseline and last age at onset, sex, and family structure (for FHS) as covariates. We further evaluated model sensitivity to fasting blood glucose and low‐density lipoprotein levels. In addition, we assessed the impact of *APOE* ε4 by comparing primary models to those with *APOE* ε4 adjustment and stratification, where sample size permitted.

### 2.5 Network‐based target prioritization and drug repurposing

#### 2.5.1 Target prioritization

Given the large number of genes per network, we applied a weighted PageRank (PR) ^26^, ^27^ algorithm to score gene importance to network structure.^26^, ^28^ We used the *igraph* R package to generate weighted PR scores for each cell‐based network using the Topological Overlap Matrix similarity scores from the WGCNA as edge.^29^, ^30^ These weights represent the likelihood of a gene interacting with another gene. Prioritized genes were those with PR scores classified as outliers (interquartile range [IQR] method) of each network's PR distribution. A binomial proportion test compared AD differentially expressed gene enrichment in prioritized versus non‐prioritized genes.

#### 2.5.2 Characterization of the top‐ranked prioritized genes

We utilized brain expression profiles of the prioritized genes using previous results from ROSMAP, MAYO, and FHS/Boston University Alzheimer's Disease Research Center (BU‐ADRC) cohorts.^15^ Bulk RNA‐seq–based differential expression and neuropathological association results were available from 568 ROSMAP (DLPFC), 162 MAYO (temporal cortex), and 208 FHS/BU‐ADRC brains. Differential expressions for top‐ranked prioritized genes were assessed against cell‐based risk status using bulk FHS/BU‐ADRC brain RNA, where quality control (QC) and normalization are described elsewhere.^15^ Limma models were fit to log2‐transformed fragments per kilobase of transcript per million mapped reads (FPKM) normalized expression values, accounting for family structure using the *duplicateCorrelation* function, and adjusted for age, sex, and RNA integrity number (RIN).

#### 2.5.3 Pathway and drug perturbation analysis with prioritized genes

We conducted pathway enrichment analysis using the *enrichr* package in R. We identified candidate drugs modulating prioritized network genes using the *enrichr* Drug_Perturbations_from_GEO_up and Drug_Perturbations_from_GEO_down gene‐set libraries, with significance defined by Enrichr's Benjamini–Hochberg–adjusted *p*‐value. Because disease directionality was derived from brain bulk RNA data and perturbation databases rely primarily on non‐brain cell lines, candidate drugs were nominated based on significant modulation of prioritized genes rather than a required match to the tissue‐level disease direction. The direction and functional consequence of each candidate drug were subsequently assessed experimentally. Finally, nominated drugs were prioritized based on prior AD/dementia clinical trial history to test our hypothesis that genetic subtyping can identify responsive patient groups and improve therapeutic outcomes.

### 2.6 Experimental validation of candidate drugs using hiPSC‐derived astrocytes

Pooled astrocyte lines of *APOE* ε3/ε3 (*APOE33*; *n* = 3, independently genome‐edited isogenic lines) and *APOE* ε4/ε4 (*APOE*44; *n* = 3, independently genome‐edited isogenic lines) genotypes derived from hiPSCs^31^ were cultured in Matrigel‐coated six‐well plates in Astrocyte medium (ScienCell: 1801, astrocyte medium [1801‐b], 2% fetal bovine serum [0010], astrocyte growth supplement [1852] and 1% Gibco Antibiotic‐Antimycotic (100X) (15240062). Cells were seeded at densities of 3 × 10^5^ cells/well of six‐well plates for the experiment and incubated in 37°C, 5% CO_2_ for 24 hours with the compounds in the following concentrations: pioglitazone [50 nM] (CDS021593, Sigma‐Aldrich, USA), β‐estradiol [0.2 nM] (E2758, Sigma‐Aldrich, USA), levetiracetam [50 ug/mL] (PHR1447, Sigma‐Aldrich, USA) and candesartan cilexetil [5 µmol/L] (SML0245, Sigma‐Aldrich, USA). The drug doses were determined based on reported in vitro toxicity and drug‐responsive studies. (1) β‐Estradiol at doses of 0.2 and 2 nM was similarly effective in protecting against Aβ‐induced toxicity.^32^ To avoid potential cytotoxicity, a lower dose of 0.2 nM was used. (2) Levetiracetam at 50 µg/mL has been shown to restore the impaired astrocyte membrane resting potentials in inflammatory in vitro co‐cultures.^33^ (3) Selected pioglitazone at a low‐dose 50 nM improves mitochondrial bioenergetics, which are compromised at a higher dose (100 nM).^34^ (4) Candesartan 5 µmol/L is proven safe and effective in reducing Lipopolysaccharide (LPS) induced inducible Nitric Oxide Synthase (iNOS) and Cyclooxygenase 2 COX‐2) expression in Blader‐Vogel 2 (BV2) cells.^35^

Total RNA was extracted from treated cells with the RNeasy kit (Cat. No. 74104, Qiagen, USA) per manufacturer instructions. RNA was quantified on a NanoDrop 2000 (Thermo Fisher Scientific, USA), and 1 µg was reverse‐transcribed to complementary DNA (cDNA) with iScript Reverse Transcription Supermix (#1708840, Bio‐Rad, USA). Comparative quantitative polymerase chain reaction (qPCR) was performed biological triplicates of cDNA samples that were differentiated from *APOE*33 and *APOE*44 lines with primers for human *C4A*, *C4B*, *APOE*, and *Glyceraldehyde‐3‐Phosphate Dehydrogenase (GAPDH*) genes using Applied Biosystems Power SYBR Green PCR Master Mix (#4367659, Applied Biosystems, USA) and ran in QuantStudio 7 Flex Real‐Time PCR System (Applied Biosystems, USA). Target‐gene expression was normalized to GAPDH and quantified by the comparative Ct method (2^−ΔΔCt^).

U.S. Food and Drug Administration (FDA)–approved drugs were administered to differentiated hiPSC‐derived astrocytes from a female AD patient carrying the *APOE* ε4/ε4 genotype (or *APOE*44), as well as the same cell line clustered regularly interspaced short palindromic repeats (CRISPR) genome edited for the isogenic human *APOE* ε3/ε3 genotype (or *APOE*33). We assessed the efficacy of the drugs in lowering the expression of *APOE*, *C4A*, and *C4B* in hiPSC‐derived astrocytes carrying *APOE*33 and *APOE*44 genotypes after treatment of the four drugs. The fold changes for each compound‐treated sample were normalized to *APOE33* vehicle control (Dimethyl Sulfoxide: DMSO) expression levels.

## 3 RESULTS

The overall study design and data sources are illustrated in Figure 1. Phase 1 generated cell‐type–specific co‐expression networks. Phase 2 defined genetic subtypes via cbPRSs and characterized them using endophenotypes and AD progression. Phase 3 prioritized genes within each network using a graph‐based algorithm and nominated repurposable drugs. Phase 4 validated the top‐ranked drugs in hiPSC‐derived astrocytes.

**FIGURE 1:**
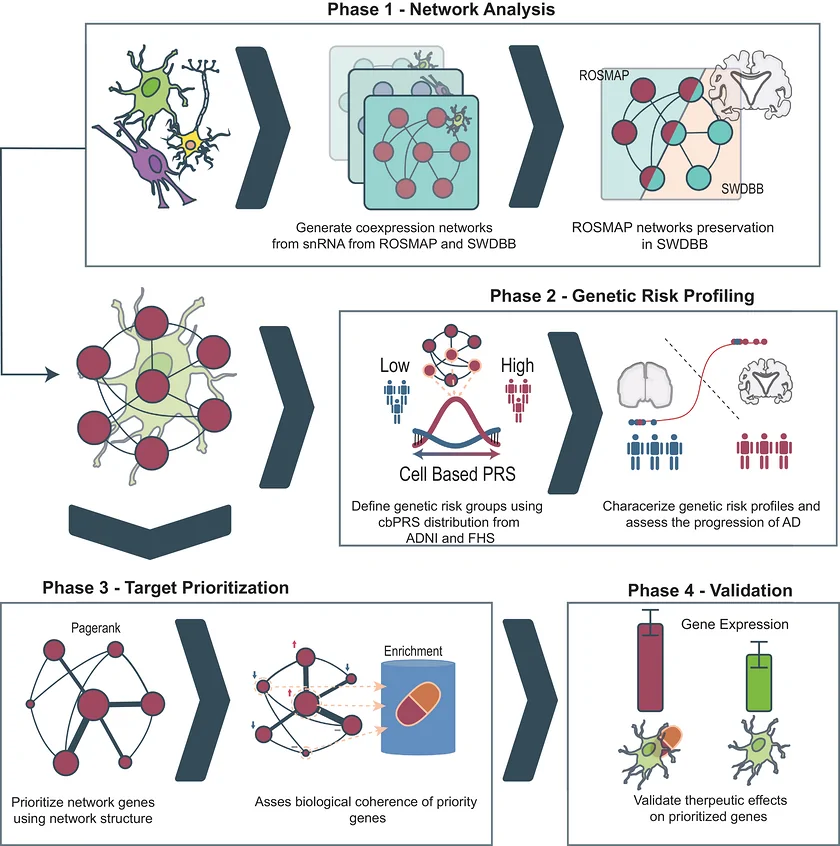
Overall study design in three phases. Phase 1 represented the process to identify brain cell type–specific co‐expression networks. Phase 2 defined and characterized genetic risk profiles. Phase 3 prioritized cell‐based targets for network perturbation. Phase 4 assessed drug efficacy in hiPSC astrocytes. AD, Alzheimer's disease; ADNI, Alzheimer's Disease Neuroimaging Initiative; cbPRS, cell‐based polygenic risk scores; FHS, Framingham Heart Study; PRS, polygenic risk score; snRNA, single‐nuclei RNA sequencing data.

### 3.1 Identified cell‐based networks and PRSs

We identified 60 co‐expression network modules in the ROSMAP dataset (e.g., astrocyte module 1: Ast‐M1). Among these, 12 modules exhibited moderate to high preservation in the SWBB dataset with Z‐summary ≥5 (Tables S1 and S2). We selected highly preserved networks with Z‐summary ≥10 for further analyses, including two astrocyte modules (Ast‐M2, Ast‐M10) and two oligodendrocyte modules (Oli‐M45, Oli‐M50) (Table 1 and Figure 2A). These modules showed no significant correlation with canonical cell markers such as *GFAP* (astrocytes) or *OLIG1/OLIG2* (oligodendrocytes) (Figure S1). To assess whether these modules reflect underlying AD diagnosis rather than cell‐type–specific co‐expression structure, we correlated donor‐level module eigengenes (aggregated across nuclei per donor) with AD/CN status across the 48 discovery donors (24 AD, 24 CN). Ast‐M2, Ast‐M10, and Oli‐M45 showed no significant eigengene–diagnosis correlation (*r* = −0.090 to 0.051, *p* = 0.54–0.83), whereas Oli‐M50 showed a non‐significant trend (*r* = 0.284, *p* = 0.050) (Table S3). Genome‐wide PRS (gwPRS) and cbPRSs were computed for 1414 ADNI participants and 8481 FHS participants (Tables S4 and S5). The ADNI cohort included 781 CN, 305 MCI, and 328 AD subjects, whereas the FHS cohort included 7631 CN, 310 MCI, and 540 AD subjects. The combined ADNI set at baseline exam contained 525 CN, 672 MCI, and 215 AD participants, whereas the number of AD cases increased to 477 participants during 3‐ to 5‐year follow‐ups. All 8310 individuals who have participated in neuropsychological examinations entered the FHS with CN at baseline. After about 40 years of monitoring with up to 13 neuropsychological evaluations, 540 participants developed AD in FHS. Mean cbPRS and gwPRS values were comparable between the ADNI and FHS cohorts (Figure S2 and Table S6). Both gwPRS (odds ratio [OR] = 1.09, *p* = 7.71 × 10^−5^) and cbPRSs from Ast‐M2 and Oli‐M45 networks (ORs > 1.22, *p* < 4.4×10^−6^) were positively associated with the conversion from MCI to AD (Tables S7 and S8). The cbPRSs outperformed gwPRS for AD progression prediction while using fewer markers.

**TABLE 1:** Characteristics of preserved and AD‐associated cell‐based networks.

PRS Network	No. Genes	Z‐summary	Estimate (AD)	FDR *p* (AD)	GWS Genes	No. SNPs	ADNI cbPRS Mean (SD)	FHS cbPRS Mean (SD)
GW (All)	NA	NA	NA	NA	All	442	50.02 (3.84)	49.53 (3.03)
Ast‐M2	1032	11.48	−9.00	4.13 × 10^−18^	*APOE, CD2AP, CLU*	65	23.38 (1.99)	22.58 (1.40)
Ast‐M10	449	10.67	3.93	6.23 × 10^−4^	*BIN1, SLC24A4*	10	0.88 (0.44)	0.86 (0.43)
Oli‐M45	1542	17.27	−3.52	1.74 × 10^−3^	*APOE, CR1, INPP5D, PTK2B, SLC24A4*	66	23.38 (1.99)	22.59 (1.40)
Oli ‐M50	1964	18.25	18.13	1.96 × 10^−71^	*ABCA7, BIN1, CLU*	15	1.29 (0.52)	1.26 (0.47)

*Note*: Polygenic risk score (PRS) using single nucleotide polymorphism (SNP) with *p* < 5.4 × 10^−8^ from genome‐wide genes (GWs) and cell type specific genes from astrocyte (Ast), oligodendrocyte (Oli), and oligodendrocyte progenitor cell (Opc). Cell‐based networks with Z‐summary scores of 10 or higher were considered preserved. Networks showing significant differences in eigenvalues between AD and control samples were considered AD‐associated networks. Networks with fewer than two genome‐wide significant genes were excluded from analysis.

Estimate for AD: t‐statistic for AD in differential eigengene analysis.

FDR *p* for AD: false discovery rate *p*‐value for AD in differential eigengene analysis.

GWS Genes: Genes contain a SNP with *p* < 5.4 × 10^−8^.

GW: genome wide PRS includes all GWS genes. No. SNPs: total number of genome‐wide significant SNPs in network.

Mean cbPRS (SD): Mean and standard deviation of the cell‐based polygenic risk score.

**FIGURE 2 alz71895-fig-0002:**
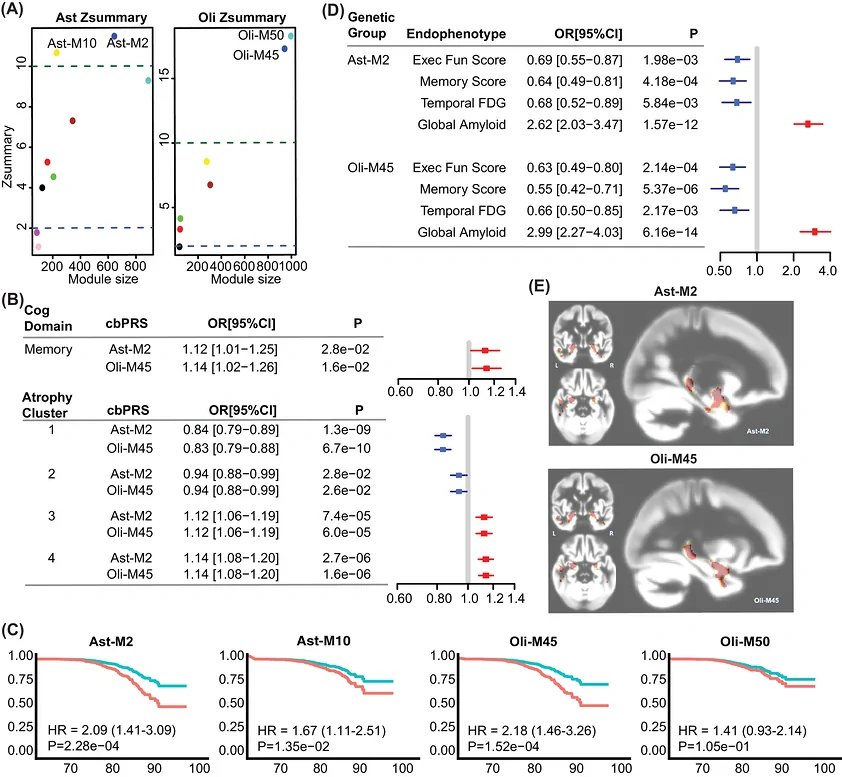
Association of genetic risk groups with AD endophenotypes and cognitive progression in ADNI and FHS. (A) Preservation of WGCNA networks for Ast and Oli. Modules with a Z summary statistic ≥10 were considered preserved. (B) Association of quantitative cbPRSs with previously defined cognitive subgroups and MRI imaging clusters in ADNI 1‐2/GO. Association of cbPRS with cognitively defined subgroups including age at last as a covariate. Association of cbPRS with MRI imaging clusters, including age at transition as an additional covariate. Cognitive associations are not survived after multiple test corrections with a Bonferroni‐corrected significance of *p* < 1.67×10^−^^3^. (C) Alzheimer's disease progression in individuals with quartile‐defined low and high risk of Ast‐M2, Ast‐M10, Oli‐M45, and Oli‐M50 in FHS. Models accounted for left‐sided censoring, with a baseline age less than 75 years and a survival age greater than or equal to 65. Survival age refers to the earliest age of AD diagnosis or censoring. Non‐focal terms were marginalized by their mean and reference values. (D) Association analysis of genetic risk status with AD endophenotypes in ADNI 1, 2/GO, three cohorts. Genetic risk status was modeled as a binary outcome in a logistic regression with MRI endophenotypes (hippocampal volume and entorhinal cortex thickness). (E) Significantly smaller brain volumes, primarily in the temporal lobes, were observed in association with genetic risk status for Ast‐M2 and Oli‐M45 after correction for multiple comparisons using a standard false discovery rate (FDR) of 5%. Gray matter volume deficits for Ast‐M2 and Oli‐M45 risk groups were detected through voxel‐wise quantitative brain analysis. Regions in red indicate significantly reduced grey matter volumes compared to the template.

To evaluate genetic independence among the four cbPRSs, we evaluated pairwise correlations and shared genomic loci across modules (Tables S9 and S10). Ast‐M2 and Oli‐M45 were nearly identical (*r* = 0.994), sharing 55 of 58–59 loci (≈93%–95%) and representing a single underlying genetic signal. Accordingly, their progression associations both attenuated after adjusting for *APOE* ε4 dosage. Conversely, Ast‐M10 and Oli‐M50 showed a moderate correlation (*r* = 0.824), with 43% of Oli‐M50 loci (6 of 14) unique from Ast‐M10. Because their associations strengthened under *APOE* adjustment, they capture a distinct, largely *APOE*‐independent signal. Consequently, we report Ast‐M2/Oli‐M45 as a single genetic risk axis with cell‐type–specific transcriptional readouts and maintain Ast‐M10 and Oli‐M50 as distinct genetic scores.

We evaluated the association of cbPRSs with previously defined cognitive subtypes (Table S11) and neuroimaging atrophy clusters (Table S12) in ADN1‐2/GO (Figure 2B). Elevated cbPRSs for the Ast‐M2 and Oli‐M45 networks increased the odds of membership to the AD–Memory cognitive subtype (OR > 1, *p* < 0.05) at a nominal significance level (Table S13). High cbPRSs for these same networks were also associated with an increased odds for brain atrophy Clusters 3 (localized temporal atrophy), 4 (advanced global atrophy), and 2 (mild diffuse atrophy), and a significantly decreased odds for Cluster 1, which represents minimal atrophy. The results for Ast‐M2 and Oli‐M45 remain statistically significant after adjusting for multiple comparisons. Although these results suggest a global atrophy association for Ast‐M2 and Oli‐M45, Ast‐M10 showed nominal significance with atrophy Cluster 3 (OR = 1.27, *p* = 4.41 × 10^−3^) (Figure 2B and Table S14).

### 3.2 Association of AD biomarkers and progression for cell‐based risk status

Participants were stratified into high‐ and low‐risk groups based on the cbPRS median and quartiles thresholds for FHS and ADNI (Tables S15–S18). Because FHS provided a longer observation window (≈40 years) than ADNI (≈4–5 years) on cognition, we focused AD progression analyses in FHS (Tables S19–S22). Since quartile‐based stratification for Ast‐M2, Ast‐M10, and Oli‐M45 produced greater group separation than median cutoffs across all networks, quartile‐defined groups were used for subsequent survival analyses (Figure 2C and Table S23). AD progression rates differed by cell‐based risk status across networks (Figure 2C). *APOE* ε4 dosage adjustment revealed a nominally significant association for Oli‐M50, which was not significant at baseline (hazard ratio [HR] = 1.25, *P* = 6.84 × 10^−^^2^ → HR_APOE _= 1.33, P_APOE _= 2.34 × 10^−^^2^), and strengthened the already‐significant association for Ast‐M10 (HR = 1.41, *P* = 6.94 × 10^−^^3^ → HR_APOE_ = 1.46, P_APOE _= 3.69×10^−^^3^), suggesting that genetic risk profiles in these subgroups are independent of the *APOE* ε4 allele. However, the signals were substantially attenuated for Ast‐M2 and Oli‐M45 (Tables S23 and S24) after *APOE* ε4 dosage adjustment. In contrast, vascular‐adjusted models yielded higher hazard estimates for AD in high‐risk groups for both Ast‐M2 (HR = 1.81, *p* = 1.56×10^−6^; HR_VAS _= 2.09, P_VAS _= 2.28×10^−4^) and Oli‐M45 (HR = 2.02, *p* = 2.56 × 10^−8^; HR_VAS _= 2.18, P_VAS _= 1.52 × 10^−4^), indicating that these networks are influenced primarily by the *APOE* ε4 allele but not by the vascular risk factor. Despite the lower rates of incident AD and shorter follow‐up data in ADNI, high‐risk status for the Ast‐M2 and Oli‐M45 networks was significantly associated with faster AD progression, even after multiple testing correction threshold at Bonferroni‐adjusted significance of *p* < 1.25 × 10^−2^ (Table S25 **)**. This attenuation pattern for Ast‐M2 and Oli‐M45 with *APOE* ε4 dosage adjustment was replicated in ADNI with reduced quartile‐based HRs by approximately half, while signals remained significant (Ast‐M2: HR = 7.31→3.41; Oli‐M45: HR = 7.58→3.31). Ast‐M10 and Oli‐M50 remained non‐significant regardless of *APOE* adjustment (Table S26).

Endophenotypes of AD including impairedmemory and executive function, lower hippocampal volume, reduced glucose metabolism, and higher global amyloid accumulation were significantly associated with cell‐based risk status of the Ast‐M2 and Oli‐M45 networks in ADNI (Bonferroni‐adjusted *p* < 2.8 × 10^−3^) (Figure 2D and Table S27). Effects of endophenotypes were strongest in MCI subjects, where the amyloid odds ratio (OR) more than doubled that of CN or AD (Table 2). Following *APOE* ε4 adjustment, high‐risk subjects continued to exhibit a nominally significant association with glucose metabolism in the Oli‐M45 network (Table S28). Voxel‐wise analysis showed that high‐risk status for Ast‐M2 and Oli‐M45 was associated with reduced gray‐matter volumes in the temporal lobe (FDR *p* = 0.0006 at *q* = 0.05; Figure 2E). Affected regions included bilateral amygdala, hippocampal head and posterior body, bilateral inferior temporal gyri, left middle temporal gyrus, and left fusiform gyrus.

**TABLE 2 alz71895-tbl-0002:** Association with AD endophenotypes using genetic risk status in ADNI.

Genetic Subtype and endophenotype	CN		MCI		AD	
	OR (95% CI)	*p*	OR (95% CI)	pi	OR (95% CI)	*p*
**Ast‐M2**						
Global Amyloid	1.61 (1.07, 2.49)	0.027	**3.72 (2.52, 5.78)**	**4.21 × 10^−10^**	1.63 (0.49, 6.84)	0.441
Temporal FDG	0.77 (0.4, 1.43)	0.418	0.69 (0.48, 0.97)	0.037	0.91 (0.27, 2.93)	0.877
Memory score	1.28 (0.79, 2.07)	0.315	**0.47 (0.29, 0.74)**	**1.44 × 10^−3^**	0.7 (0.12, 3.44)	0.665
Entorhinal cortex thickness	1.33 (0.81, 2.21)	0.265	1.11 (0.84, 1.49)	4.54E‐01	0.07 (0, 0.43)	0.032
**Oli‐M45**						
Global amyloid	1.99 (1.29, 3.19)	0.003	**3.72 (2.5, 5.85)**	**1.12 × 10^−9^**	1.37 (0.29, 8.93)	0.699
Temporal FDG	0.86 (0.47, 1.52)	0.608	0.65 (0.45, 0.93)	0.020	0.92 (0.26, 3.07)	0.894
Memory score	1.08 (0.67, 1.74)	0.745	**0.41 (0.25, 0.66)**	**3.01 × 10^−4^**	3.76 (0.45, 55.18)	0.250
Executive function score	0.67 (0.41, 1.05)	0.084	0.68 (0.47, 0.99)	0.045	4.44 (1.01, 39.15)	0.097
Entorhinal cortex thickness	1.5 (0.92, 2.51)	0.112	1.09 (0.82, 1.47)	0.551	0.03 (0, 0.34)	0.043

Abbreviations: AD, Alzheimer's disease cases; CN, cognitively normal subjects; MCI, mild cognitive impairment subjects.

Odds ratio (OR) and 95% confidence interval (95% CI) were obtained from association tests of endophenotypes using genetic subtype status as a binary outcome in a logistic regression. Bold *p* values indicated tests passing false discovery rate (FDR) *p* < 2.08 × 10^−3^.

### 3.3 Network‐based target prioritization identified key regulatory hubs in AD

Graph‐based prioritization reduced network[Fig alz71895-fig-0002] size by up to 98% (Figure 3A) while enriching differentially expressed genes (DEGs) between control and AD brains (Table S29 and Figure 3B). The proportion of DEGs in the prioritized gene sets increased from 16% to 62%, with the Ast‐M2 and Oli‐M50 networks containing more than 50% DEGs after selection. Pathway enrichment indicated immune, neurodegenerative, and metabolic pathways across networks (Figure S3). Prioritized genes from the Ast‐M2 network were primarily enriched for pathways related to prion disease and Parkinson's disease, whereas those from the Oli‐M45 network were linked to AD and amyotrophic lateral sclerosis. Both Ast‐M2 and Oli‐M45 shared enrichment for Parkinson's disease, coronavirus disease, and ribosome pathways. Ast‐M10 was uniquely enriched for vascular risk factors and comorbidities (diabetes, thyroid disease), and Oli‐M50 was involved in actin cytoskeleton regulation.

**FIGURE 3 alz71895-fig-0003:**
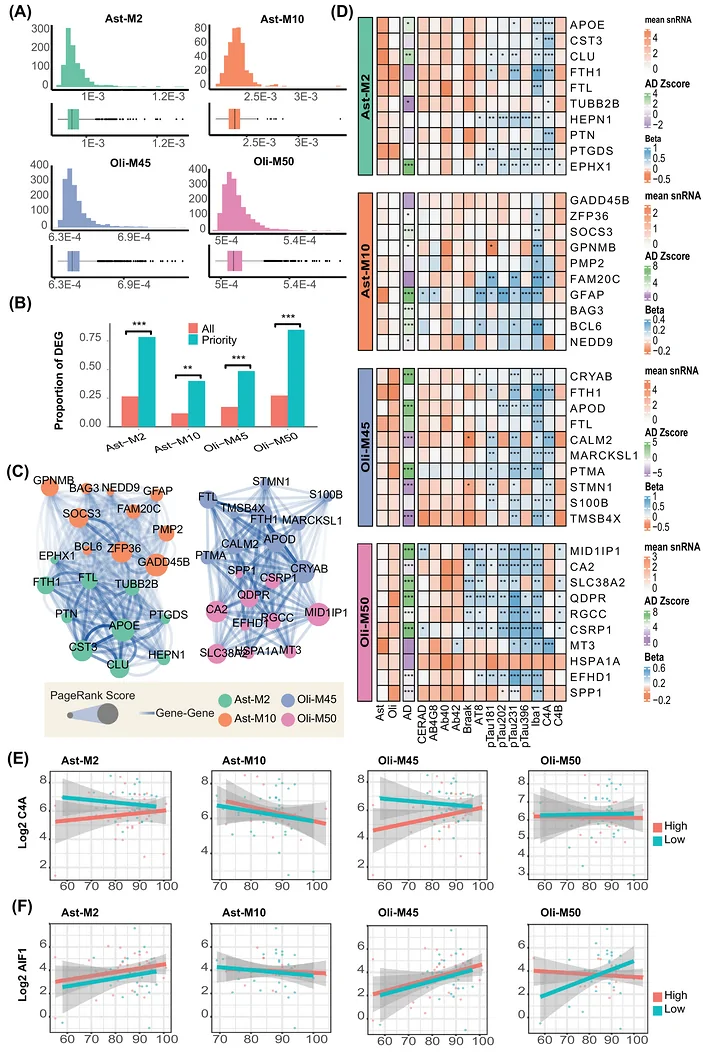
Target prioritization using the network centrality and functional enrichment of prioritized genes in AD. (A) Network centrality distribution for networks. The distribution displayed a highly skewed pattern, highlighting outlier genes with significantly increased importance to the overall network structure. (B) Binomial proportion tests comparing the proportion of differentially expressed genes for AD between priority and non‐prioritized genes. (C) Gene–gene interaction network of top 10 ranked genes. The edge color indicates the topological overlap matrix (TOM) value from the WGCNA analysis, scaled from weakest to strongest similarity for each cell type. Node size corresponded to the gene's network‐specific centrality score. (D) Expression profiles of the top 10 centrality‐prioritized genes (top to bottom), highlighting cell‐specific expression patterns and their association with neuropathological traits. (E) Log‐linear regression plots demonstrating the expression of *C4A* over time for genetic subgroups. Models were adjusted for sex, family structure, and RIN. All non‐focal terms were marginalized using their mean or reference value.

The top 10 prioritized genes per network formed dense interaction hubs in both astrocytes and oligodendrocytes (Figure 3C). These genes were consistently upregulated in AD versus control brain cells (Figure 3D). The prioritized genes for Ast‐M2 and Oli‐M45 networks were significantly correlated with increased microglial activation (Iba1) and complement protein C4a (Figure 3D). Oli‐M50 showed the strongest associations with Braak stage and phosphorylated tau (p‐tau), although no network showed consistent associations with amyloid pathology (Figure 3D).

We examined *C4A* and *Iba1* transcripts in cbPRS‐derived risk groups from bulk RNA sequencing data of FHS postmortem brains (Table S30). *C4A* results mirrored the protein‐level analysis in FHS (Table S31). High‐risk status for the Ast‐M2 and Oli‐M45 networks was associated with significantly higher *C4A* expression, whereas the Ast‐M10 and Oli‐M50 networks showed no difference between high‐risk and low‐risk groups (Figure 3E). These results indicate specific complement‐pathway involvement for Ast‐M2 and Oli‐M45 networks. In contrast, *AIF1* encoding Iba1 protein did not differ significantly between high‐risk and low‐risk groups for any of the four networks (Table S31). This suggests that protein‐level microglial activation is not captured by bulk‐tissue *AIF1* transcription.

### 3.4 Identified candidate drugs targeting top‐ranked networks

Enrichr analysis of prioritized genes identified 51, 50, and 50 significantly associated compounds for the Ast‐M2, Oli‐M45, and Oli‐M50 networks, respectively (Tables S32–S34).[Table alz71895-tbl-0002] Data mining with network‐based targets in pharmacological databases identified seven candidate drugs with prior AD clinical‐trial history and known capacity to perturb *APOE* expression, a key genetic driver of AD for Ast‐M2 and Oli‐M45 networks (Table S35). Of these, four drugs including estradiol, levetiracetam, pioglitazone, and candesartan showed documented modulation of *APOE* expression in cell lines and perturbed top‐ranked prioritized genes in both the Ast‐M2 (Figures 4A and 4B) and Oli‐M45 networks (Figure S4). These four drugs were selected as candidates modulating target‐drug interactions in astrocytes.

**FIGURE 4 alz71895-fig-0004:**
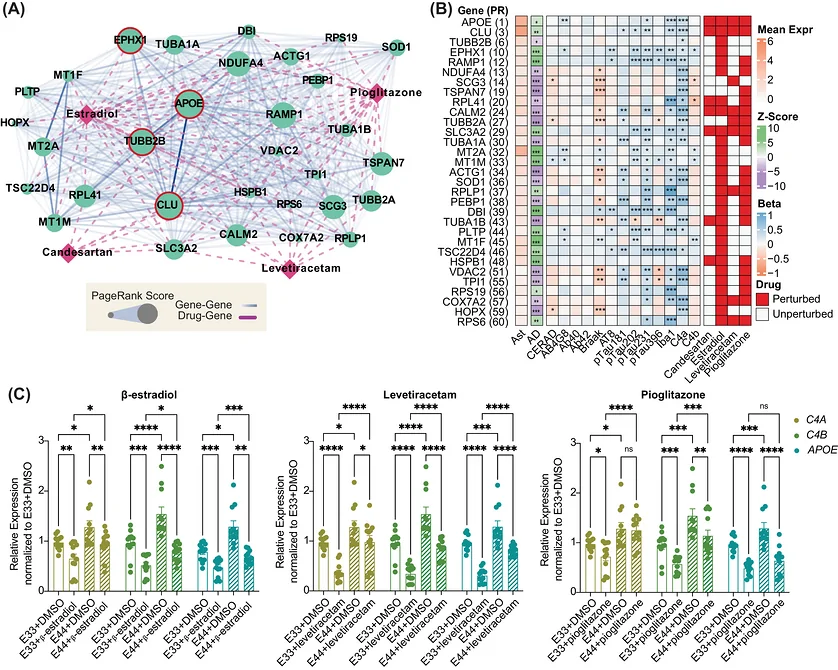
Gene–drug interaction network and validation of prioritized gene‐perturbating compounds for the Ast‐M2 network. (A) Gene–drug interaction network constructed from prioritized genes perturbed by top‐ranked drugs. Edge color indicates topological overlap matrix (TOM) value from the WGCNA analysis, scaled from weakest to strongest similarity for each cell type. Node size corresponded to the gene's network‐specific centrality score, with genes ranked within the top 10 for the Ast‐M2 outlined in red. (B) Expression profiles of centrality‐prioritized genes perturbed by top‐ranked drugs. These profiles displayed cell‐specific expressions with distinct biological pathways associated with prioritized genes. (C) Efficacy of drug treatments in reducing the expression of *C4A*, *C4B*, and *APOE* in *APOE33 and APOE44* human iPSC‐derived astrocytes. Relative gene expression of *C4A*, *C4B*, and *APOE* was measured in *APOE33* and *APOE44* hiPSC‐derived astrocytes before and after treatment with β‐estradiol (left), levetiracetam (middle), and pioglitazone (right). Gene expression was assessed by RT‐qPCR and presented as fold change relative to the DMSO vehicle control in *APOE33* astrocytes. Error bar represented the standard error of the mean. Statistics: *n* = 4 biological replicates (wells) per genotype and treatment condition, each with three technical replicates from a total of two independent differentiations from a pooled isogenic iPSC‐astrocyte line per *APOE* genotype. Excluded ∆Ct > 0.5 cycles from the mean of the other technical replicates as outliers. Two‐way ANOVA, *p ≤ 0.05, **p ≤ 0.01, ***p ≤ 0.001, and ****p ≤ 0.0001.

### 3.5 Validation of network‐prioritized drugs in hiPSC‐derived astrocytes

To experimentally validate the effects of *APOE*‐targeted drugs, we treated isogenic hiPSC‐derived astrocyte lines of *APOE ε3/ε3 (APOE33)* or *APOE ε4/ε4 (APOE44)* with four candidate compounds. After 24 hours of treatment, no significant cytotoxicity or impaired cell proliferation was observed (Figure S5). To evaluate reference‐gene stability for qPCR normalization, *GAPDH* and *Actin Beta (ACTB)* stability were assessed using the geNorm algorithm. *GAPDH* showed acceptable stability across biological replicates (expression stability value [M‐value] range: 0.3–1.5) with no significant cycle threshold (Ct) differences between drug treatment and vehicle control in either genotype. ACTB, by contrast, showed gene instability in candesartan‐treated *APOE44* cells (*M* = 1.69) and significant Ct changes in levetiracetam‐treated *APOE33* cells; *GAPDH* was therefore used as the reference gene for all qPCR analyses (Figure S6). Consistent with the expected AD patient pathology, *APOE44* astrocytes exhibited significantly higher baseline expressions of *C4A*, *C4B*, and *APOE* compared with *APOE*33 astrocytes (Figure 4C, Figure S7). β‐Estradiol and levetiracetam emerged as the most potent compounds, significantly reducing the expression of *C4A*, *C4B*, and *APOE* by more than twofold (*p* < 0.01) across both *APOE* genotypes. On average, β‐estradiol reduced target‐gene expression by 37.52% in *APOE33* astrocytes and 41.32% in *APOE44* astrocytes. Pioglitazone lowered C4A expression in *APOE33* astrocytes but lost efficacy in *APOE44* astrocytes (Figure 4C), failing to significantly reduce *APOE* expression. Candesartan failed to lower the target‐gene expression in both *APOE33* and *APOE44* astrocytes (Figure S8). These results show that prioritized astrocyte network components are functionally modifiable, supporting the biological coherence of the identified risk‐associated networks.[Fig alz71895-fig-0003]

## 4 DISCUSSION

Our cellular network–based framework using cbPRSs identified two genetic risk axes underlying AD heterogeneity: an *APOE*‐concentrated axis, reflected in both an astrocyte (Ast‐M2) and an oligodendrocyte (Oli‐M45) co‐expression network, and a second, an *APOE*‐independent axis reflected in Ast‐M10 and Oli‐M50. These subtypes demonstrated strong associations with hippocampal volume deficits, decreased glucose metabolism, and global amyloid deposition. Amyloid accumulation begins during normal aging and accelerates through preclinical and early symptomatic AD, including MCI.^36^, ^37^ Consistent with this, high‐risk individuals at cell‐level showed elevated amyloid deposition at the MCI stage and faster cognitive decline. Covariate‐adjusted analyses confirmed that genetic effects for Ast‐M2 and Oli‐M45 networks were influenced by the *APOE* ε4 dosage but not by vascular risk factors, whereas those for Ast‐M10 and Oli‐M50 were independent from both *APOE* ε4 dosage and vascular risk factors. These findings may be particularly relevant for individuals who develop AD without the *APOE* ε4 allele, highlighting the value of cell‐based stratification beyond traditional *APOE* genotyping. Because Ast‐M2 and Oli‐M45 share the substantial majority of their constituent risk loci, their converging phenotypic associations most likely reflect one underlying *APOE*‐region genetic signal manifesting through two distinct cell‐type–specific disease mechanisms. These are evidenced by the divergent pathways of their prioritized hub genes. Given the well‐established role of *APOE* as a risk factor for both cerebrovascular disease and AD,^38^ our results illustrate complicated interactions between *APOE*‐related cellular pathways and vascular risk factors. Together, these results underscore that our cell‐based PRS framework can disentangle different disease mechanisms underlying AD heterogeneity.[Fig alz71895-fig-0004]

Robust cell‐based subtypes are particularly useful for AD prevention and treatment, linking genetic risk to disease pathology.^6^, ^39^ Cell‐specific analyses clarify how risk variants drive cellular dysfunction, and recent work on *APOE* genotypes^15^, ^31^, ^40^, ^41^ and their associations with AD biomarkers^42^ have elucidated our understanding of AD pathology. Most recently, network‐based PRSs offer greater power and are more discriminative than traditional gwPRSs.^43^ Network‐based PRS approaches have demonstrated improved ability to explain disease heterogeneity and identify distinct mechanisms, both in AD^9^, ^43^ and in other complex diseases such as Type 2 diabetes.^44^

We used a graph‐based PR algorithm to simplify complex network interactions and prioritize candidate genes. The algorithm identified *APOE* as the top node within the dense Ast‐M2 network. Elevated apoE protein levels disrupt the immunomodulatory functions of astrocytes, which serve as the brain's primary apoE producers and key immune regulators.^31^, ^45^ Expression profiles of the prioritized genes further confirm the importance of complement‐mediated neuroinflammation for the Ast‐M2 and Oli‐M45 subtypes. Our findings reinforce previous reports of an *APOE* genotype–dependent mechanism linking to expression levels of *C4A* and *C4B* in AD.^15^, ^40^ Reactive glia co‐localize with complement around amyloid plaques,^46^ and complement proteins can act on astrocytes by releasing pro‐inflammatory cytokines, leading to neuronal death and glial activation.^46^ Oligodendrocytes provide trophic support to central nervous system (CNS) axons via myelination, and myelin dysregulation contributes to AD‐related neuropathology.^47^ The Oli‐M45 network was associated with volume alterations in regions central to memory circuits via entorhinal–neocortical connections.

We validated four candidate drugs targeting the *APOE*‐centered network (Ast‐M2) using the hiPSC‐derived astrocytes. Estradiol and levetiracetam exhibited the most potent effect on *APOE*, *C4A*, and *C4B* expression, whereas pioglitazone demonstrated an *APOE* genotype–dependent effect, being effective only in *APOE33* astrocytes. Previous studies confirmed anti‐inflammatory and neuroprotective effects through modulation of complement components for estradiol^48^, ^49^ and pioglitazone.^50^, ^51^, ^52^ Estrogen therapies such as estradiol can have *APOE* genotype–dependent effect on *APOE* expression^53^ and induce pro‐myelinating effects and promote maturation in oligodendrocytes.^54^, ^55^ Levetiracetam is an anticonvulsant that upregulates *PTGDS* (ranked 9 in Ast‐M2), which regulates anti‐inflammatory roles in astrocytes^56^, ^57^ and is involved in amyloid metabolism.^58^, ^59^ A recent clinical trial demonstrated levetiracetam improved executive functioning and spatial memory in AD cases with subclinical epileptiform,^60^ suggesting that proper subgrouping of AD cases may improve levetiracetam's efficacy. Pioglitazone, an anti‐diabetic medication, has gained interest as an AD therapeutic by inhibiting proinflammatory genes through Peroxisome Proliferator‐Activated Receptor Gamma (PPARγ) activation.^50^, ^61^ Clinical trial results indicate improved cognitive function in patients with diabetes^62^ but failed to replicate neuroprotective effects in non‐diabetic populations.^63^, ^64^, ^65^ Our experimental validation for pioglitazone using hiPSC‐derived astrocytes illustrated improved efficacy in astrocytes carrying the *APOE* ε3/ε3 genotype, highlighting its *APOE* genotype–specific effectiveness in astrocytes.

We recognize several limitations in our study. First, the modest sample size used to generate the co‐expression networks contributed to larger network sizes and increased sensitivity to parameter selection. In addition, specific cell types, including microglia, were underrepresented in the snRNA‐seq data and were either excluded or filtered due to low reproducibility. Furthermore, because the co‐expression networks were constructed from a case–control pooled discovery sample, we cannot fully rule out that downstream enrichment for AD‐associated differential expression partially reflects the same disease‐driven transcriptional variation that indirectly shaped network structure, even though diagnosis was not used as an explicit variable in module construction or selection. Donor‐level module eigengene–diagnosis correlations for Ast‐M2, Ast‐M10, and Oli‐M45 were not significant (Table S3), suggesting that these modules are not primarily organized around AD diagnosis. We acknowledge partial sample overlaps between our 48 discovery brains and the ROSMAP bulk RNA‐seq differential expression dataset used for enrichment testing. The overlapping brains (*n* = 48) represent a small fraction (8.5%) of the 568‐brain ROSMAP bulk cohort. Crucially, two downstream findings including postmortem C4A protein associations (Figure 3D) and target validation in hiPSC‐derived astrocytes (Figure 4C) were completely independent of RNA‐seq results and free of sample overlap with network construction. Second, all samples were from prefrontal cortex, although AD has region‐specific effects on brain physiology and protein expression.^66^ Focusing on a single region may have limited our ability to identify more distinct cellular subtypes. Third, AD GWAS summary statistics from European‐ancestry studies may limit generalizability to diverse populations, and our focus on genome‐wide significant variants missed broader contributions to AD phenotypic variance. In addition, pairwise correlations among the four cbPRSs revealed substantial genomic overlap between Ast‐M2 and Oli‐M45 (55 of 58–59 shared loci) and, to a lesser extent, between Ast‐M10 and Oli‐M50 (8 of 9–14 shared loci). Our *APOE*‐adjusted analyses addressing this redundancy are currently limited to the progression outcomes in FHS and ADNI (Table S26). Further evaluation is needed to understand the impact of *APOE* genotypes on endophenotypes, cognitive subtypes, or VBM analyses in ADNI. Fourth, we used an undirected weighted PR with an IQR cutoff. Although PR is typically applied to directed graphs, its undirected form imperfectly reflects degree distribution,^67^ and we did not enforce directionality because drug‐perturbation data came largely from non‐brain cells. Still, the enriched proportion of known AD genes in the priority sets supports the approach. Although this approach avoided excluding viable candidates, it requires empirical validation in cell‐type models such as our hiPSC‐derived astrocyte assays to establish true disease reversal. Fifth, restricting drug prioritization to compounds with a history of AD/dementia clinical trials prioritizes translational feasibility and the potential to rescue previously unsuccessful agents via genetic stratification, but intentionally excludes novel, unstudied therapeutic candidates. Finally, the hiPSC‐astrocyte validation did not account for cell–cell interactions or modulation by other cell types, and one individual genetic background was assessed. The *APOE* ε4/ε4 impairs neuronal electrical activity by altering oligodendrocyte cholesterol deposition,^68^ so the observed *APOE*‐dependent effects cannot be fully explained without co‐culture validation.

## 5 CONCLUSION

We developed and applied a novel cellular network–based approach for genetic subtyping and targeted repurposing in AD. By integrating genetic and transcriptomic data, we identified distinct cellular subtypes and prioritized drugs that modulate key AD‐related pathways in brain cells. Our findings highlight the potential of cbPRSs in predicting disease progression and i*n vitro* perturbation therapeutic responses. The experimental validation of candidate drugs using hiPSC‐derived astrocytes provided a foundation of initial functional validation for precision medicine approaches in AD treatment. Future studies incorporating co‐culture systems and more comprehensive datasets are essential to fully elucidate the therapeutic potential of these drugs across different cellular contexts and patient populations. Our approach advances the understanding of AD pathology and offers new avenues for targeted therapeutic interventions.

## CONFLICT OF INTEREST STATEMENT

The authors declare no conflicts of interest. Author disclosures are available in the Supporting Information.

## CONSENT STATEMENT

The study protocol, design, and performance of the current study were approved by the Boston University Institutional Review Board.

## Supporting information




Supporting Information

Supporting Information

Supporting Information

Supporting Information

Supporting Information
