# Supplementary material for: Cell‐based polygenic risk scores predict clinical progression and prioritize network‐based therapeutic targets in Alzheimer's disease
Source: Alzheimers Dement. 2026 Oct 6;22(10):e71895. doi: 10.1002/alz.71895 (PMC13640970; doi:10.1002/alz.71895)
Supplement: Supplementary file 2 [file ALZ-22-e71895-s001.docx]

Supplementary Information

Supplementary Table 1. Cell count from single-nuclei RNA sequencing data.

| Cell Type | ROSMAP | | |  | SWDBB | | |
| --- | --- | --- | --- | --- | --- | --- | --- |
|  | **Total** | **AD** | **CN** |  | **Total** | **AD** | **CN** |
| Ast | 3392 | 1830 | 1562 |  | 16095 | 7547 | 8548 |
| End | 116 | 56 | 60 |  | 1810 | 462 | 1348 |
| Ex | 34768 | 17822 | 16946 |  | 50428 | 24909 | 25519 |
| In | 7847 | 3840 | 4007 |  | 20233 | 10244 | 9989 |
| Mic | 1000 | 489 | 511 |  | 6285 | 3112 | 3173 |
| Oli | 18235 | 9035 | 9200 |  | 38985 | 17162 | 21823 |
| Opc | 2626 | 1289 | 1337 |  | 10128 | 4931 | 5197 |

**ROSMAP**: Religious Orders Study and Rush Memory and Aging Project; **SWDBB**: Southwest Dementia Brain Bank; **AD**: number of cells from Alzheimer Disease samples; **CN**: number of cells from cognitively normal samples.

Supplementary Table 2. Networks moderately preserved at the z-summary > 5.

| **Network** | | **No. Genes** | **Z-summary** |  |
| --- | --- | --- | --- | --- |
| **Ast -M2** | **1032** | | **11.48** | |
| Ast -M8 | 273 | | 5.26 | |
| Ast -M9 | 1500 | | 9.28 | |
| **Ast -M10** | **449** | | **10.67** | |
| Ex-M15 | 134 | | 7.58 | |
| Ex-M17 | 5190 | | 9.24 | |
| Ex-M18 | 46 | | 8.75 | |
| **Oli-M45** | **1542** | | **17.27** | |
| Oli-M46 | 548 | | 6.74 | |
| **Oli-M50** | **1964** | | **18.25** | |
| Oli-M51 | 532 | | 8.55 | |
| Opc-M53 | 1087 | | 6.23 | |

A z-summary score of 10 or higher was considered highly preserved and were followed for downstream analysis. Highly preserved networks are bolded.

**Supplementary Table 3.** Correlation between module eigengene and AD diagnosis in the snRNA-seq discovery cohort.

| **Module** | **Cell type** | **N donors (AD/CN)** | **Mean eigengene (AD)** | **Mean eigengene (CN)** | **r** | **P** |
| --- | --- | --- | --- | --- | --- | --- |
| Ast-M2 | Astrocyte | 24/24 | 0.0007 | 0.0035 | -0.090 | 0.542 |
| Ast-M10 | Astrocyte | 24/24 | 0.0045 | 0.0027 | 0.051 | 0.728 |
| Oli-M45 | Oligodendrocyte | 24/24 | 0.0001 | -0.0003 | 0.031 | 0.834 |
| Oli-M50 | Oligodendrocyte | 24/24 | 0.0023 | -0.0014 | 0.284 | 0.050 |

Supplementary Table 4. Sample size with GWAS data in ADNI at last exam.

| **DX Last** | **ADNI 1-2/GO** | | | |  | **ADNI 1-3** | | | |
| --- | --- | --- | --- | --- | --- | --- | --- | --- | --- |
|  | **N** | **N with ε4+** | **% Female** | **Mean Age at Last (sd)** |  | **N** | **N with ε4+** | **% Female** | **Mean Age at Last (sd)** |
| CN | 557 | 212 | 42.00 | 80.10 (7.34) |  | 781 | 287 | 46.4 | 77.8 (7.85) |
| MCI | 256 | 100 | 40.60 | 78.50 (7.89) |  | 305 | 122 | 39.7 | 77.8 (8.1) |
| AD | 310 | 205 | 44.80 | 78.10 (7.77) |  | 328 | 219 | 44.5 | 77.9 (7.89) |

**ADNI:** the Alzheimer’s Disease Neuroimaging Initiative (ADNI); **DX Last**: AD diagnosis at last exam; **AD**: Alzheimer’s disease subjects; **CN**: cognitively normal control subjects not diagnosed with AD or MCI; **MCI**: mild cognitive impairment subjects; **N**: number of subjects; **ε4+**: **ε4** carriers; % **Female**: percentage of female. **Mean Age at Last**: mean (standard deviation, sd) age at last exam.

Supplementary Table 5. Sample size with GWAS data in FHS at last exam.

| **Dx Last** | **N** | **N with ε4+** | **% Female** | **Mean Age at Last (sd)** |
| --- | --- | --- | --- | --- |
| CN | 7,631 | 1,530 | 46.80 | 65.57 (14.46) |
| MCI | 310 | 76 | 45.81 | 77.38 (11.25) |
| AD | 540 | 170 | 32.78 | 84.37 (7.56) |

**FHS**: Framingham Heart Study; **DX Last**: AD diagnosis at last exam; **AD**: Alzheimer’s disease subjects; **CN**: cognitively normal control subjects with no AD dementia or MCI; **MCI**: mild cognitive impairment subjects; **N**: number of subjects; **ε4+**: **ε4** carriers; % **Female**: percentage of female. **Mean Age at Last**: mean (standard deviation, sd) age at last exam.

Supplementary Table 6. Summary of cell-based polygenic risk scores

| **PRS** | **Mean cbPRS (sd)** | |
| --- | --- | --- |
|  | **ADNI 1-3** | **FHS** |
| GW | 50.02 (3.84) | 49.53 (3.03) |
| Ast-M2 | 23.38 (1.99) | 22.58 (1.40) |
| Ast-M10 | 0.88 (0.44) | 0.86 (0.43) |
| Oli-M45 | 23.38 (1.99) | 22.59 (1.40) |
| Oli -M50 | 1.29 (0.52) | 1.26 (0.47) |

Polygenic risk score (**PRS**) using single nucleotide polymorphism (SNP) with p<5.4x10^-8^ from genome-wide genes (GW) and cell type specific genes from astrocyte (Ast), oligodendrocyte (Oli), and oligodendrocyte progenitor cell (Opc).

**Mean cbPRS (sd):** Mean and standard deviation of the cell based polygenic risk score using SNPs within the gene region (+-50k bases). SNPs were collected from AD GWAS summary statistics (Kunkle et al., 2019) with P<5.4x10^-8^, minor allele frequency (>1%), imputation quality (R^2^>0.4), and linkage disequilibrium (r^2^ < 0.5).

Supplementary Table 7. Characterization of converted subjects in the combined ADNI cohort.

| **Progression** | **N** | **%Female** | **Mean Age at Last (sd)** | **Mean PRS (sd)** | | | | |
| --- | --- | --- | --- | --- | --- | --- | --- | --- |
|  |  |  |  | **GW** | **Ast-M2** | **Ast-M10** | **Oli-M45** | **Oli-M50** |
| CN to MCI | 35 | 40.0 | 84.87 (5.02) | 50.1 (3.8) | 23.32 (2.07) | 0.85 (0.41) | 23.29 (2.08) | 1.27 (0.54) |
| CN to AD | 15 | 60.0 | 85.94 (4.45) | 50.61 (4.38) | 23.67 (1.9) | 0.96 (0.32) | 23.6 (1.95) | 1.31 (0.49) |
| MCI to AD | 143 | 41.3 | 78.36 (7.45) | 51.07 (3.83) | 24.02 (1.9) | 0.87 (0.41) | 24.03 (1.89) | 1.30 (0.49) |

AD progression was defined as diagnosis change between at baseline and at last exam; standard deviation (sd).

Supplementary Table 8. Association of genome-wide and cell-based PRSs with AD progression in the combined ADNI cohort.

| **PRS** | **CN to MCI** | |  | **CN to AD** | |  | **MCI to AD** | |
| --- | --- | --- | --- | --- | --- | --- | --- | --- |
|  | **OR (95% CI)** | **P** |  | **OR (95% CI)** | **P** |  | **OR (95% CI)** | **P** |
| GW | 1.03 (0.93, 1.13) | 5.50E-01 |  | 1.09 (0.94, 1.26) | 2.28E-01 |  | 1.09 (1.05, 1.13) | 7.71E-05 |
| Ast-M2 | 1.02 (0.84, 1.22) | 8.40E-01 |  | 1.18 (0.89, 1.55) | 2.30E-01 |  | 1.22 (1.12, 1.22) | 4.41E-06 |
| Ast-M10 | 1.01 (0.46, 2.16) | 9.87E-01 |  | 1.76 (0.55, 5.44) | 3.27E-01 |  | 0.92 (0.61, 2.16) | 6.76E-01 |
| Oli-M45 | 1.01 (0.84, 1.21) | 9.05E-01 |  | 1.16 (0.87, 1.52) | 2.91E-01 |  | 1.23 (1.12, 1.21) | 3.48E-06 |
| Oli-M50 | 1.02 (0.51, 2.01) | 9.48E-01 |  | 1.16 (0.4, 3.25) | 7.80E-01 |  | 1.03 (0.73, 2.01) | 8.78E-01 |

CN: cognitively normal; MCI: mild cognitively impaired; OR: odds ratio; CI: confidence interval; P: nominal significance.

Progression status was defined using the diagnosis at baseline and diagnosis at last. Logistic regression using progression status as outcome was adjusted for age at last and sex.

**Supplementary Table 9.** Pairwise correlation among cell-based polygenic risk scores.

| **Cell-based PRS** | **Ast-M2** | **Ast-M10** | **Oli-M45** | **Oli-M50** |
| --- | --- | --- | --- | --- |
| **Ast-M2** | 1.000 | 0.028 | 0.994 | 0.051 |
| **Ast-M10** | 0.028 | 1.000 | 0.034 | 0.824 |
| **Oli-M45** | 0.994 | 0.034 | 1.000 | 0.033 |
| **Oli-M50** | 0.051 | 0.824 | 0.033 | 1.000 |

**Supplementary Table 10.** Shared genomic loci between cell-based polygenic risk score (PRS) modules.

| **Cell-based PRS** | **Ast-M2** | **Ast-M10** | **Oli-M45** | **Oli-M50** |
| --- | --- | --- | --- | --- |
| **Ast-M2** | 58 | 0 | 55 | 2 |
| **Ast-M10** | 0 | 9 | 1 | 8 |
| **Oli-M45** | 55 | 1 | 58 | 0 |
| **Oli-M50** | 2 | 8 | 0 | 14 |

Supplementary Table 11. Sample distribution of cognitively defined AD subtypes in ADNI 1-2/GO.

| **Cognitive Subtype** | **N** | **%Female** | **Mean Age at Last (sd)** |
| --- | --- | --- | --- |
| Executive | 16 | 38.5 | 76.9 (8.0) |
| Language | 48 | 33.3 | 78.1 (6.9) |
| Memory | 184 | 43.5 | 77.8 (7.0) |
| Multiple | 25 | 40.0 | 75.7 (6.0) |
| No domain | 275 | 42.2 | 76.8 (8.0) |
| Visuospatial | 85 | 38.8 | 75.3 (9.3) |

**N**: subgroup sample size (Mukherjee et al., 2020). **Mean Age at Last**: age at last screening expressed as mean; standard deviation: sd.

Supplementary Table 12. Sample distribution of brain atrophy clusters in ADNI 1-2/GO.

| **Brain Atrophy Cluster** | **Characteristics of brain atrophy and cognitive decline** | **N** | **%Female** | **ε4+** | **Mean Age at Last (sd)** |
| --- | --- | --- | --- | --- | --- |
| 1 | preserved brain volume exhibits no significant atrophy across the brain compared to CN | 738 | 46.4 | 391 | 71.8 (7.4) |
| 2 | mild diffuse atrophy, with widespread mild cortical atrophy without pronounced medial temporal lobe atrophy | 561 | 41.9 | 394 | 74.4 (8.0) |
| 3 | focal medial temporal lobe atrophy, showing localized atrophy in the hippocampus and the anterior-medial temporal cortex with relative sparing elsewhere | 490 | 45.1 | 542 | 74.9 (7.2) |
| 4 | advanced atrophy, displaying severe atrophy over the whole brain including severe temporal lobe atrophy. | 668 | 41.6 | 540 | 76.2 (7.2) |

Distribution includes total counts from AD and control subjects from the combined ADNI-1, ADNI-GO, ADNI-2 datasets. Image patterns are treated independently as individuals may transition between image atrophy patterns (. Four distinct clusters were identified: cluster 1 exhibits normal neuroanatomical profiles with the slowest progression; cluster 2 exhibits mild diffuse atrophy; cluster 3 exhibits temporal atrophy; cluster 4 exhibits advanced atrophy over the whole brain. **Mean Age at Last**: age at last screening expressed as mean (standard deviation).

**Supplementary Table 13**. Association of quantitative cbPRSs and gwPRS for previously defined cognitive subgroups in the combined ADNI cohort.

| **Cognitive Subtype** | **PRS** | **OR [95% CI]** | **P** |
| --- | --- | --- | --- |
| No Domain | GWS | 1.02 [0.97, 1.07]] | 4.96E-01 |
|  | Ast-M2 | 0.99 [0.9, 1.09] | 8.33E-01 |
|  | AST-M10 | 1.33 [0.85, 2.08] | 2.07E-01 |
|  | OLI-M45 | 0.99 [0.9, 1.09] | 8.00E-01 |
|  | OLI-M50 | 1.17 [0.8, 1.7] | 4.22E-01 |
| Visuospatial | GWS | 0.95 [0.88, .03] | 2.15E-01 |
|  | AST-M2 | 0.91 [0.78, 1.05] | 2.05E-01 |
|  | AST-M10 | 0.7 [0.34, 1.39] | 3.12E-01 |
|  | OLI-M45 | 0.9 [0.78, 1.05] | 1.85E-01 |
|  | OLI-M50 | 0.83 [0.46, 1.48] | 5.44E-01 |
| Memory | GWS | 1.05 [0.99, 1.11] | 8.91E-02 |
|  | AST-M2 | 1.12 [1.01, 1.25] | 2.70E-02 |
|  | AST-M10 | 0.87 [0.54, 1.39] | 5.57E-01 |
|  | OLI-M45 | 1.13 [1.02, 1.26] | 1.80E-02 |
|  | OLI-M50 | 1.07 [0.71, 1.59] | 7.57E-01 |
| Executive function | GWS | 0.98 [0.84, .13] | 7.38E-01 |
|  | AST-M2 | 0.99 [0.74, 1.31] | 9.56E-01 |
|  | AST-M10 | 0.97 [0.25, 3.51] | 9.61E-01 |
|  | OLI-M45 | 0.98 [0.73, 1.29] | 8.63E-01 |
|  | OLI-M50 | 0.71 [0.22, 2.15] | 5.65E-01 |
| Multiple | GWS | 0.87 [0.74, 1.01] | 8.28E-02 |
|  | AST-M2 | 0.82 [0.61, 1.08] | 1.73E-01 |
|  | AST-M10 | 1.54 [0.44, 5.29] | 4.90E-01 |
|  | OLI-M45 | 0.8 [0.59, 1.06] | 1.36E-01 |
|  | OLI-M50 | 1.15 [0.39, 3.19] | 7.89E-01 |
| Language | GWS | 0.94, [0.85, 1.03] | 1.82E-01 |
|  | AST-M2 | 0.92 [0.77, 1.1] | 3.82E-01 |
|  | AST-M10 | 0.81 [0.35, 1.83] | 6.12E-01 |
|  | OLI-M45 | 0.93 [0.77, 1.11] | 4.07E-01 |
|  | OLI-M50 | 0.67 [0.31, 1.35] | 2.75E-01 |

Results are not significant after multiple testing correction (P < 1.67x10^-3^).

**PRS**: polygenic risk score; **OR**: odds ratio; **CI**: confidence interval; **P**: p value

**Supplementary Table 14.** Association of quantitative cbPRSs for previously defined neuroimaging subgroups in the combined ADNI cohort.

| **Atrophy Subgroup** | **PRS** | **OR [95% CI]** | **P** |
| --- | --- | --- | --- |
| 1 | GWS | **0.95 [0.93, 0.97]** | **4.77E-06** |
|  | AST-M2 | **0.88 [0.85, 0.91]** | **3.98E-11** |
|  | AST-M10 | 0.93 [0.78, 1.09] | 3.57E-01 |
|  | OLI-M45 | **0.88 [0.85, 0.91]** | **2.55E-11** |
|  | OLI-M50 | 1.05 [0.90, 1.21] | 5.41E-01 |
| 2 | GWS | 0.98 [0.95, 1.00] | 3.07E-02 |
|  | AST-M2 | **0.94 [0.91, 0.98]** | **8.61E-04** |
|  | AST-M10 | 0.84 [0.71, 0.99] | 3.49E-02 |
|  | OLI-M45 | **0.94 [0.91, 0.98]** | **1.34E-03** |
|  | OLI-M50 | 0.87 [0.75, 1.00] | 5.30E-02 |
| 3 | GWS | **1.05 [1.02, 1.07]** | **1.03E-04** |
|  | AST-M2 | **1.09 [1.06, 1.13]** | **7.23E-07** |
|  | AST-M10 | 1.27 [1.08, 1.49] | 4.41E-03 |
|  | OLI-M45 | **1.09 [1.05, 1.13]** | **9.67E-07** |
|  | OLI-M50 | 1.13 [0.97, 1.30] | 1.08E-01 |
| 4 | GWS | 1.03 [1.01, 1.06] | 2.82E-03 |
|  | AST-M2 | **1.10 [1.06, 1.14]** | **1.63E-07** |
|  | AST-M10 | 1.02 [0.87, 1.20] | 8.16E-01 |
|  | OLI-M45 | **1.10 [1.06, 1.14]** | **1.58E-07** |
|  | OLI-M50 | 0.98 [0.85, 1.14] | 8.22E-01 |

Bold P values indicate tests passing false wise error rate (FWER) (P < 1.67x10^-3^).

**PRS**: polygenic risk score; **OR**: odds ratio; **CI**: confidence interval; **P**: p value

Supplementary Table 15. Sample characteristics for quartile genetic groups in FHS.

| **Network** | **DX Last** | **Low (Status=0)** | | | |  | | **High (Status=1)** | | |
| --- | --- | --- | --- | --- | --- | --- | --- | --- | --- | --- |
|  |  | **N** | **Female** | **ε4+** | **Mean Aget at Last (sd)** |  | **N** | **Female** | **ε4+** | **Mean Aget at Last (sd)** |
| M2 | AD | 116 | 68.1 | 8 | 85.09 (6.86) |  | 184 | 67.39 | 107 | 83.19 (7.18) |
|  | CN | 1887 | 54.05 | 95 | 65.76 (14.52) |  | 1805 | 52.8 | 972 | 65.69 (14.2) |
|  | MCI | 62 | 54.84 | 5 | 76.09 (12.63) |  | 93 | 52.69 | 52 | 75.67 (12.67) |
| M10 | AD | 113 | 63.72 | 31 | 84.89 (8.2) |  | 143 | 67.83 | 42 | 83.52 (8.14) |
|  | CN | 1880 | 53.67 | 390 | 65.45 (14.53) |  | 1850 | 52.76 | 377 | 65.29 (14.05) |
|  | MCI | 76 | 59.21 | 20 | 77.58 (12.47) |  | 87 | 51.72 | 22 | 77.46 (9.86) |
| M45 | AD | 110 | 67.27 | 9 | 84.89 (6.89) |  | 188 | 67.02 | 108 | 83.14 (7.21) |
|  | CN | 1890 | 53.44 | 90 | 66 (14.6) |  | 1804 | 53.05 | 972 | 65.45 (14.17) |
|  | MCI | 63 | 55.56 | 6 | 75.82 (12.77) |  | 91 | 54.95 | 49 | 76.4 (11.92) |
| M50 | AD | 119 | 64.71 | 41 | 85.24 (8.03) |  | 142 | 66.9 | 40 | 83.62 (7.95) |
|  | CN | 1878 | 52.93 | 390 | 65.49 (14.47) |  | 1858 | 52.21 | 386 | 65.55 (14.49) |
|  | MCI | 78 | 62.82 | 16 | 76.8 (12.54) |  | 78 | 53.85 | 19 | 76.69 (10.24) |

FHS subjects stratified risk by quartiles such that the 1^st^ quartile and 4^th^ quartile define low and high risk**. DX Last:** last diagnosis. **N**: number of subjects with diagnosis in strata. **High**: cbPRSs in the 4^th^ quartile. **Low**: cbPRSs in the 1^st^ quartile. **Mean Age at Last**: age at last screening expressed as mean and standard deviation (sd).

Supplementary Table 16. Sample characteristics for median genetic groups in FHS.

| **Network** | **DX Last** | **Low (Status=0)** | | | |  | | **High (Status=1)** | | |
| --- | --- | --- | --- | --- | --- | --- | --- | --- | --- | --- |
|  |  | **N** | **Female** | **ε4+** | **Mean Aget at Last (sd)** |  | **N** | **Female** | **ε4+** | **Mean Aget at Last (sd)** |
| M2 | AD | 228 | 66.67 | 29 | 84.68 (7.68) |  | 312 | 67.63 | 141 | 84.14 (7.47) |
|  | CN | 3778 | 53.04 | 260 | 65.46 (14.56) |  | 3682 | 53.34 | 1270 | 65.68 (14.35) |
|  | MCI | 142 | 54.93 | 12 | 77.51 (11.18) |  | 168 | 53.57 | 64 | 77.27 (11.35) |
| M10 | AD | 248 | 64.52 | 83 | 84.79 (7.59) |  | 292 | 69.52 | 87 | 84.01 (7.52) |
|  | CN | 3751 | 53.27 | 763 | 65.53 (14.54) |  | 3709 | 53.11 | 767 | 65.61 (14.38) |
|  | MCI | 155 | 56.77 | 36 | 77.85 (12.46) |  | 155 | 51.61 | 40 | 76.91 (9.92) |
| M45 | AD | 224 | 67.86 | 23 | 84.66 (7.74) |  | 316 | 66.77 | 147 | 84.16 (7.43) |
|  | CN | 3789 | 53.34 | 257 | 65.54 (14.54) |  | 3671 | 53.04 | 1273 | 65.6 (14.38) |
|  | MCI | 138 | 55.07 | 11 | 77.98 (11.12) |  | 172 | 53.49 | 65 | 76.9 (11.37) |
| M50 | AD | 249 | 63.86 | 86 | 84.76 (7.69) |  | 291 | 70.1 | 84 | 84.03 (7.44) |
|  | CN | 3762 | 53.22 | 778 | 65.52 (14.51) |  | 3698 | 53.16 | 752 | 65.62 (14.41) |
|  | MCI | 149 | 58.39 | 36 | 77.71 (11.15) |  | 161 | 50.31 | 40 | 77.07 (11.38) |

FHS subjects stratified such that risk below and above the median define low and high risk**. DX Last:** last diagnosis. **N**: number of subjects with diagnosis in strata. **High**: cbPRSs in the 4^th^ quartile. **Low**: cbPRSs in the 1^st^ quartile. **Mean Age at Last**: age at last screening expressed as mean and standard deviation (sd).

Supplementary Table 17. Sample characteristics of quartile genetic risk groups in the combined ADNI cohort.

| **Network** | **DX Last** | **Low-Risk (status=0)** | | | |  | **High-Risk (Status=1)** | | | |
| --- | --- | --- | --- | --- | --- | --- | --- | --- | --- | --- |
|  |  | **N** | **Female** | **ε4+** | **Mean Age at Last (sd)** |  | **N** | **Female** | **ε4+** | **Mean Age at Last (sd)** |
| M2 | AD | 78 | 32.05 | 3 | 77.76 (9.22) |  | 183 | 35.52 | 179 | 75.58 (7.4) |
|  | CN | 77 | 49.35 | 2 | 77.29 (7.02) |  | 41 | 48.78 | 34 | 75.81 (8.8) |
|  | MCI | 87 | 40.23 | 4 | 78.45 (8.02) |  | 69 | 43.48 | 67 | 74.27 (7.26) |
| M10 | AD | 126 | 35.71 | 78 | 78 (7.34) |  | 129 | 44.96 | 83 | 75.94 (8.15) |
|  | CN | 79 | 46.84 | 23 | 78.15 (7.37) |  | 64 | 43.75 | 24 | 74.76 (8.01) |
|  | MCI | 62 | 35.48 | 21 | 79.87 (7.87) |  | 81 | 43.21 | 35 | 75.6 (9.21) |
| M45 | AD | 75 | 30.67 | 4 | 77.56 (9.35) |  | 185 | 35.14 | 181 | 75.67 (7.26) |
|  | CN | 85 | 48.24 | 2 | 76.78 (7.13) |  | 42 | 45.24 | 34 | 76.65 (9.05) |
|  | MCI | 87 | 40.23 | 2 | 78.91 (8.07) |  | 70 | 45.71 | 69 | 74.08 (7.27) |
| M50 | AD | 129 | 34.88 | 90 | 78.19 (6.93) |  | 129 | 41.86 | 85 | 76.33 (8.28) |
|  | CN | 64 | 48.44 | 17 | 78.97 (6.67) |  | 65 | 46.15 | 23 | 75.48 (8.32) |
|  | MCI | 69 | 40.58 | 29 | 79.19 (7.93) |  | 79 | 37.97 | 31 | 76.1 (9.1) |

ADNI subjects stratified by quartiles such that the 1^st^ quartile and 4^th^ quartile define low and high risk**. DX Last:** last diagnosis. **N**: number of subjects with diagnosis in strata. **High**: cbPRSs in the 4^th^ quartile. **Low**: cbPRSs in the 1^st^ quartile. **Mean Age at Last**: age at last screening expressed as mean and standard deviation (sd).

Supplementary Table 18. Sample characteristics of median genetic risk groups in the combined ADNI cohort.

| **Network** | **DX Last** | **Low-Risk (status=0)** | | | |  | **High-Risk (Status=1)** | | | |
| --- | --- | --- | --- | --- | --- | --- | --- | --- | --- | --- |
|  |  | **N** | **Female** | **ε4+** | **Mean Age at Last (sd)** |  | **N** | **Female** | **ε4+** | **Mean Age at Last (sd)** |
| M2 | AD | 174 | 40.8 | 30 | 78.26 (8.61) |  | 344 | 39.24 | 309 | 76.01 (7.28) |
|  | CN | 157 | 50.96 | 10 | 77.6 (6.85) |  | 97 | 52.58 | 65 | 76.44 (8.34) |
|  | MCI | 169 | 38.46 | 17 | 78.98 (8.06) |  | 136 | 41.18 | 105 | 76.3 (7.94) |
| M10 | AD | 249 | 40.16 | 156 | 77.15 (7.65) |  | 269 | 39.41 | 183 | 76.41 (7.97) |
|  | CN | 140 | 49.29 | 41 | 78.04 (7.11) |  | 114 | 54.39 | 34 | 76.07 (7.76) |
|  | MCI | 148 | 37.16 | 57 | 78.41 (7.47) |  | 157 | 42.04 | 65 | 77.2 (8.64) |
| M45 | AD | 167 | 39.52 | 30 | 78.15 (8.69) |  | 351 | 39.89 | 309 | 76.11 (7.29) |
|  | CN | 161 | 51.55 | 12 | 77.52 (6.85) |  | 93 | 51.61 | 63 | 76.53 (8.41) |
|  | MCI | 167 | 38.32 | 15 | 78.89 (7.98) |  | 138 | 41.3 | 107 | 76.45 (8.08) |
| M50 | AD | 249 | 38.55 | 159 | 77.4 (7.7) |  | 269 | 40.89 | 180 | 76.18 (7.89) |
|  | CN | 133 | 51.88 | 36 | 78.33 (6.86) |  | 121 | 51.24 | 39 | 75.87 (7.89) |
|  | MCI | 150 | 39.33 | 58 | 78.81 (7.61) |  | 155 | 40 | 64 | 76.79 (8.46) |

ADNI subjects stratified such that risk below and above the median define low and high risk**. DX Last:** last diagnosis. **N**: number of subjects with diagnosis in strata. **High**: cbPRSs in the 4^th^ quartile. **Low**: cbPRSs in the 1^st^ quartile. **Mean Age at Last**: age at last screening expressed as mean and standard deviation (sd).

Supplementary Table 19. Survival sample characteristics for quartile risk groups in FHS.

|  | **Low Risk** | | | |  | **High Risk** | | | |
| --- | --- | --- | --- | --- | --- | --- | --- | --- | --- |
|  | **Ast-M2** | **Ast-M10** | **Oli-M45** | **Oli-M50** |  | **Ast-M2** | **Ast-M10** | **Oli-M45** | **Oli-M50** |
| **N** | 1178 | 1158 | 1187 | 1158 |  | 1233 | 1163 | 1227 | 1178 |
| **Incident AD** | 115 | 110 | 109 | 116 |  | 180 | 139 | 184 | 139 |
| **Female (%)** | 55.5 | 55.7 | 55.3 | 56.1 |  | 56.4 | 53.4 | 56.7 | 53.9 |
| **+ε4 (%)** | 5.4 | 23.2 | 5.3 | 23.3 |  | 55.5 | 21.9 | 55.3 | 21.5 |
| **Baseline Age (sd)** | 39.2 (10.6) | 39.4 (10.3) | 39.3 (10.8) | 39.2 (10.1) |  | 38.9 (9.9) | 39.1 (10.3) | 39.1 (10) | 39 (10.3) |
| **Follow-up (sd)** | 39.5 (12.2) | 39.4 (12.3) | 39.4 (12.2) | 39.5 (12.4) |  | 39.3 (11.7) | 39.3 (12) | 39 (11.9) | 39.5 (11.9) |
| **Conversion Age (sd)** | 85.4 (6.2) | 85.6 (7.2) | 85.2 (6.2) | 86 (6.4) |  | 83.6 (6.4) | 84.3 (6.8) | 83.5 (6.4) | 84.2 (6.8) |
| **Glucose (sd)** | 105.5 (23.1) | 105.4 (23.2) | 105.3 (23.1) | 106.1 (25.5) |  | 104.9 (23.1) | 106.6 (24.9) | 105.4 (24) | 106.5 (23.8) |
| **LDL (sd)** | 101.1 (31.9) | 102.9 (32.2) | 101.8 (32.1) | 101.7 (31.9) |  | 104.3 (33.1) | 102.9 (34.3) | 103.6 (33.2) | 103.7 (34.4) |

Summary table of genotyped subjects with longitudinal clinical data. **Incident** **AD**: number of converted cases; **BL Age**: mean age at first exam; **Follow-up** : mean follow up time; **Conversion**: mean age of conversion. **+ε4**: APOE ε4 carrier; **LDL**: Low-density Lipoprotein; **Glucose**: Fasting Blood Glucose.

Supplementary Table 20. Survival sample characteristics for median risk groups in FHS.

| **Characteristics** | **Low Risk** | | | |  | **High Risk** | | | |
| --- | --- | --- | --- | --- | --- | --- | --- | --- | --- |
|  | **Ast-M2** | **Ast-M10** | **Oli-M45** | **Oli-M50** |  | **Ast-M2** | **Ast-M10** | **Oli-M45** | **Oli-M50** |
| **N** | 2315 | 2347 | 2323 | 2343 |  | 2413 | 2381 | 2405 | 2385 |
| **Incident AD** | 224 | 244 | 220 | 244 |  | 307 | 287 | 311 | 287 |
| **Female (%)** | 55.6 | 56.4 | 55.9 | 55.6 |  | 55.5 | 54.8 | 55.3 | 55.6 |
| **+ε4 (%)** | 7.6 | 22.6 | 7.3 | 23.4 |  | 36.6 | 22.2 | 37.1 | 21.4 |
| **Baseline Age (sd)** | 39.2 (10.5) | 39.2 (10.2) | 39.3 (10.6) | 39.2 (10.2) |  | 38.9 (9.9) | 39 (10.3) | 38.9 (9.9) | 38.9 (10.2) |
| **Follow-up (sd)** | 39.5 (12.2) | 39.5 (12.1) | 39.3 (12.1) | 39.4 (12.2) |  | 39.6 (11.8) | 39.5 (11.8) | 39.7 (11.8) | 39.7 (11.8) |
| **Conversion Age (sd)** | 85.2 (6.7) | 85.2 (6.7) | 85.2 (6.7) | 85.2 (6.9) |  | 84.5 (6.9) | 84.4 (6.8) | 84.5 (6.8) | 84.4 (6.7) |
| **Glucose (sd)** | 106 (23.4) | 105.5 (24.3) | 105.7 (23) | 105.9 (24.5) |  | 105.7 (25.2) | 106.2 (24.4) | 106 (25.6) | 105.8 (24.2) |
| **LDL (sd)** | 102.3 (32.5) | 103.2 (32.2) | 102.2 (32.5) | 102.9 (32.4) |  | 104.1 (33.6) | 103.2 (34) | 104.2 (33.6) | 103.6 (33.8) |

Summary table of genotyped subjects with longitudinal clinical data. **Incident AD**: number of converted cases; **BL Age**: mean age at first exam; **Follow-up** : mean follow up time; **Conversion**: mean age of conversion. **+ε4**: APOE ε4 carrier; **LDL**: Low-density Lipoprotein; **Glucose**: Fasting Blood Glucose.

Supplementary Table 21. Survival sample characteristics for quartile risk groups in ADNI.

| **Characteristics** | **Low Risk** | | | |  | **High Risk** | | | |
| --- | --- | --- | --- | --- | --- | --- | --- | --- | --- |
|  | **Ast-M2** | **Ast-M10** | **Oli-M45** | **Oli-M50** |  | **Ast-M2** | **Ast-M10** | **Oli-M45** | **Oli-M50** |
| **N** | 98 | 103 | 105 | 104 |  | 136 | 120 | 137 | 118 |
| **Incident AD** | 10 | 36 | 10 | 41 |  | 70 | 33 | 69 | 36 |
| **Female (%)** | 39.8 | 37.9 | 41.9 | 38.5 |  | 44.1 | 41.7 | 43.8 | 41.5 |
| **e4+ (%)** | 3.1 | 46.6 | 1 | 49 |  | 96.3 | 50.8 | 96.4 | 53.4 |
| **Baseline Age (SD)** | 70 (3.8) | 70.1 (3.2) | 69.9 (3.7) | 70.2 (3.6) |  | 69.3 (3.7) | 69.5 (3.5) | 69.5 (3.6) | 69.3 (3.6) |
| **Follow-up (SD)** | 5.1 (3.5) | 4.4 (2.8) | 5.1 (3.5) | 4.7 (3.2) |  | 3.5 (2.8) | 4.1 (3.5) | 3.6 (2.8) | 4 (3.4) |
| **Conversion Age (SD)** | 76.6 (3.9) | 73.7 (3.1) | 76.6 (3.9) | 73.7 (3.7) |  | 72.7 (4.1) | 73.6 (5.1) | 73 (3.9) | 73.7 (5.3) |

Summary table of genotyped subjects with longitudinal clinical data. **Incident AD**: number of converted cases; **BL Age**: mean age at first exam; **Follow-up**: mean follow-up time; **Conversion**: mean age of conversion. **+ε4**: APOE ε4 carrier; **LDL**: Low-density Lipoprotein; **Glucose**: Fasting Blood Glucose.

Supplementary Table 22. Survival sample characteristics for median risk groups in ADNI.

| **Characteristics** | **Low Risk** | | | |  | **High Risk** | | | |
| --- | --- | --- | --- | --- | --- | --- | --- | --- | --- |
|  | **Ast-M2** | **Ast-M10** | **Oli-M45** | **Oli-M50** |  | **Ast-M2** | **Ast-M10** | **Oli-M45** | **Oli-M50** |
| **N** | 204 | 228 | 206 | 209 |  | 237 | 213 | 235 | 232 |
| **Incident AD** | 35 | 77 | 36 | 69 |  | 110 | 68 | 109 | 76 |
| **Female (%)** | 42.2 | 41.2 | 44.7 | 42.6 |  | 45.6 | 46.9 | 43.4 | 45.3 |
| **e4+ (%)** | 10.3 | 47.8 | 11.7 | 46.9 |  | 85.2 | 53.5 | 84.7 | 53.9 |
| **Baseline Age (SD)** | 70 (3.6) | 70 (3.7) | 69.9 (3.6) | 70.1 (3.7) |  | 69.7 (3.5) | 69.7 (3.4) | 69.8 (3.5) | 69.7 (3.4) |
| **Follow-up (SD)** | 4.9 (3.3) | 4.6 (3.1) | 4.8 (3.4) | 4.7 (3.2) |  | 3.8 (3.1) | 4 (3.4) | 3.9 (3.1) | 3.9 (3.3) |
| **Conversion Age (SD)** | 75.3 (4.5) | 73.6 (3.5) | 74.9 (4.5) | 73.6 (3.8) |  | 73 (3.9) | 73.5 (4.8) | 73.1 (3.9) | 73.4 (4.5) |

Summary table of genotyped subjects with longitudinal clinical data. **Incident AD**: number of converted cases; **BL Age**: mean age at first exam; **Follow-up** : mean follow up time; **Conversion**: mean age of conversion. **+ε4**: APOE ε4 carrier; **LDL**: Low-density Lipoprotein; **Glucose**: Fasting Blood Glucose.

Supplementary Table 23. Cox proportional hazard ratio for cell-based genetic risk status in FHS.

| **Network** | **Quartile** | |  | **Median** | |
| --- | --- | --- | --- | --- | --- |
|  | **HR (95% CI)** | **P** |  | **HR (95% CI)** | **P** |
| Ast-M2 | **1.81 (1.42-2.31)** | **1.56E-06** |  | **1.44 (1.2-1.74)** | **9.18E-05** |
| Ast-M10 | **1.41 (1.1-1.8)** | **6.94E-03** |  | 1.2 (1.01-1.42) | 3.63E-02 |
| Oli-M45 | **2.02 (1.58-2.59)** | **2.56E-08** |  | **1.48 (1.25-1.76)** | **8.68E-06** |
| Oli-M50 | 1.25 (0.98-1.59) | 6.84E-02 |  | 1.18 (1-1.39) | 4.67E-02 |

Cox Proportional Hazard Ratios with 95% confidence interval were computed for high-risk subjects using low as reference. Survival models were adjusted for age and sex. **HR**: Cox Proportional Hazard Ratio. **Quartile**: risk stratification by quartiles such that the 1^st^ quartile and 4^th^ quartile define low and high risk. **Median**: risk stratification by median such that risk below and above the median define low and high risk. Bold P values indicate tests passing false wise error rate (FWER) (P < 1.25x10^-2^).

**Supplementary Table** 24. Cox proportional hazard ratio adjusted by *APOE* ε4 dosage and vascular risk factors in FHS.

| **Network** | **+*APOE*** | |  | **+LDL+Glucose** | |
| --- | --- | --- | --- | --- | --- |
|  | **HR (95% CI)** | **P** |  | **HR (95% CI)** | **P** |
| Ast-M2 | 1.29 (0.98-1.69) | 7.08E-02 |  | **2.09 (1.41-3.09)** | **2.28E-04** |
| Ast-M10 | **1.46 (1.13-1.89)** | **3.69E-03** |  | 1.67 (1.11-2.51) | 1.35E-02 |
| Oli-M45 | 1.44 (1.08-1.93) | 1.42E-02 |  | **2.18 (1.46-3.26)** | **1.52E-04** |
| Oli-M50 | 1.33 (1.04-1.7) | 2.34E-02 |  | 1.41 (0.93-2.14) | 1.05E-01 |

Cox Proportional Hazard Ratio with 95% confidence interval computed using the low-risk subgroup as a reference. **HR**: Cox Proportional Hazard Ratio; **LDL**: Low-density Lipoprotein; **Glucose**: Fasting Blood Glucose. Bold P values indicate tests passing false wise error rate (FWER) (P < 1.25x10^-2^).

Supplementary Table 25. Cox proportional hazard ratio for cell-based risk status in ADNI.

| **Network** | **Quartile** | |  | **Median** | |
| --- | --- | --- | --- | --- | --- |
|  | **HR (95% CI)** | **P** |  | **HR (95% CI)** | **P** |
| Ast-M2 | **7.31 (3.74-14.3)** | **5.93E-09** |  | **3.5 (2.39-5.14)** | **1.35E-10** |
| Ast-M10 | 0.93 (0.57-1.53) | 7.87E-01 |  | 1.08 (0.78-1.49) | 6.60E-01 |
| Oli-M45 | **7.58 (3.88-14.8)** | **3.13E-09** |  | **3.23 (2.22-4.72)** | **1.16E-09** |
| Oli-M50 | 0.95 (0.6-1.51) | 8.37E-01 |  | 1.21 (0.87-1.68) | 2.61E-01 |

Bold P values indicate tests passing false wise error rate (FWER) (P < 1.25x10^-2^).

**Supplementary Table 26**. Cox proportional hazard ratio adjusted by *APOE* ε4 dosage in ADNI.

| **Network** | **HR (95% CI)** | **P** |
| --- | --- | --- |
| Ast-M2 | **3.41 (1.41-8.26)** | **6.52E-03** |
| Ast-M10 | 0.83 (0.51-1.35) | 4.52E-01 |
| Oli-M45 | **3.31 (1.35-8.13)** | **8.93E-03** |
| Oli-M50 | 0.82 (0.52-1.3) | 3.97E-01 |

Cox Proportional Hazard Ratio with 95% confidence interval computed using the low-risk subgroup as a reference. **HR**: Cox Proportional Hazard Ratio. Bold P values indicate tests passing false wise error rate (FWER) (P < 1.25x10^-2^).

Supplementary Table 27. Association with AD endophenotypes for cell-based genetic risk status defined by median and quartile cutoffs using all subjects in the combined ADNI cohort.

| **Network** | **Endophenotype** | **Quartile** | |  | **Median** | |
| --- | --- | --- | --- | --- | --- | --- |
|  |  | **OR (95% CI)** | **P** |  | **OR (95% CI)** | **P** |
| Ast-M2 | Global Amyloid | **2.62 (2.03, 3.47)** | **1.57E-12** |  | **1.78 (1.51, 2.10)** | **9.45E-12** |
|  | Temporal FDG | **0.68 (0.52, 0.89)** | **5.84E-03** |  | **0.72 (0.60, 0.87)** | **7.47E-04** |
|  | Memory Score | **0.64 (0.49, 0.81)** | **4.18E-04** |  | **0.68 (0.57, 0.81)** | **1.80E-05** |
|  | Executive Function Score | **0.69 (0.55, 0.87)** | **1.98E-03** |  | **0.74 (0.62, 0.87)** | **2.49E-04** |
|  | Hippocampal Volume | 0.86 (0.69, 1.06) | 1.64E-01 |  | 0.88 (0.75, 1.03) | 1.07E-01 |
|  | Entorhinal Cortex Thickness | 0.90 (0.73, 1.13) | 3.69E-01 |  | 0.92 (0.79, 1.07) | 2.61E-01 |
| Ast-M10 | Global Amyloid | 1.07 (0.87, 1.32) | 5.32E-01 |  | 1.00 (0.86, 1.16) | 9.96E-01 |
|  | Temporal FDG | 0.82 (0.62, 1.08) | 1.59E-01 |  | 0.96 (0.80, 1.15) | 6.36E-01 |
|  | Memory Score | 0.87 (0.69, 1.10) | 2.35E-01 |  | 1.03 (0.87, 1.22) | 7.51E-01 |
|  | Executive Function Score | 0.88 (0.70, 1.10) | 2.58E-01 |  | 1.00 (0.86, 1.18) | 9.53E-01 |
|  | Hippocampal Volume | 0.90 (0.72, 1.12) | 3.51E-01 |  | 0.97 (0.83, 1.14) | 7.45E-01 |
|  | Entorhinal Cortex Thickness | 1.03 (0.83, 1.28) | 7.72E-01 |  | 1.09 (0.94, 1.27) | 2.72E-01 |
| Oli-M45 | Global Amyloid | **2.99 (2.27, 4.03)** | **6.16E-14** |  | **1.86 (1.58, 2.21)** | **4.73E-13** |
|  | Temporal FDG | **0.66 (0.50, 0.85)** | **2.17E-03** |  | **0.73 (0.60, 0.87)** | **8.76E-04** |
|  | Memory Score | **0.55 (0.42, 0.71)** | **5.37E-06** |  | **0.66 (0.55, 0.78)** | **3.68E-06** |
|  | Executive Function Score | **0.63 (0.49, 0.80)** | **2.14E-04** |  | **0.75 (0.63, 0.88)** | **4.49E-04** |
|  | Hippocampal Volume | 0.85 (0.68, 1.05) | 1.35E-01 |  | 0.86 (0.73, 1.00) | 5.70E-02 |
|  | Entorhinal Cortex Thickness | 0.89 (0.71, 1.11) | 3.16E-01 |  | 0.92 (0.79, 1.07) | 2.57E-01 |
| Oli-M50 | Global Amyloid | 1.11 (0.90, 1.37) | 3.38E-01 |  | 1.07 (0.92, 1.24) | 3.83E-01 |
|  | Temporal FDG | 0.90 (0.69, 1.17) | 4.36E-01 |  | 0.99 (0.82, 1.19) | 8.91E-01 |
|  | Memory Score | 1.00 (0.80, 1.25) | 9.92E-01 |  | 0.94 (0.79, 1.11) | 4.43E-01 |
|  | Executive Function Score | 1.01 (0.81, 1.27) | 9.26E-01 |  | 0.98 (0.83, 1.14) | 7.72E-01 |
|  | Hippocampal Volume | 1.02 (0.82, 1.26) | 8.82E-01 |  | 1.00 (0.85, 1.17) | 9.90E-01 |
|  | Entorhinal Cortex Thickness | 1.14 (0.92, 1.43) | 2.36E-01 |  | 1.07 (0.92, 1.25) | 3.86E-01 |

MRI endophenotypes (hippocampal volume and entorhinal cortex thickness) included intracranial volume and magnetic field strength as covariates. Associations passing multiple testing correction are bolded P < 2.8x10^-3^. **OR (95% CI):** odds ratio with 95% lower and upper bound. Genetic risk-group membership was treated as a binary outcome in the logistic regression model. **Quartile**: risk stratification by quartiles such that the 1^st^ quartile and 4^th^ quartile define low and high risk. **Median**: risk stratification by median such that risk below and above the median define low and high risk.

**Supplementary Table 28.** Association with AD endophenotypes for cell-based genetic risk quartile status using all subjects in the combined ADNI cohorts.

| **Network** | **Endophenotype** | **ε4 Dosage Unadjusted** | |  | **ε4 Dosage Adjusted** | |
| --- | --- | --- | --- | --- | --- | --- |
|  |  | **OR (95% CI)** | **P** |  | **OR (95% CI)** | **P** |
| Ast-M2 | Global Amyloid | **2.62 (2.03, 3.47)** | **1.57E-12** |  | 0.72 (0.39, 1.27) | 2.61E-01 |
|  | Temporal FDG | **0.68 (0.52, 0.89)** | **5.84E-03** |  | 1.94 (0.95, 4.1) | 7.50E-02 |
|  | Memory Score | **0.64 (0.49, 0.81)** | **4.18E-04** |  | 1.54 (0.85, 2.86) | 1.62E-01 |
|  | Executive Function Score | **0.69 (0.55, 0.87)** | **1.98E-03** |  | 0.93 (0.54, 1.61) | 7.83E-01 |
|  | Hippocampal Volume | 0.86 (0.69, 1.06) | 1.64E-01 |  | 1.39 (0.83, 2.4) | 2.19E-01 |
|  | Entorhinal Cortex Thickness | 0.9 (0.73, 1.13) | 3.69E-01 |  | 1.54 (0.88, 2.78) | 1.37E-01 |
| Ast-M10 | Global Amyloid | 1.07 (0.87, 1.32) | 5.32E-01 |  | 0.98 (0.78, 1.23) | 8.57E-01 |
|  | Temporal FDG | 0.82 (0.62, 1.08) | 1.59E-01 |  | 0.86 (0.65, 1.14) | 2.88E-01 |
|  | Memory Score | 0.87 (0.69, 1.1) | 2.35E-01 |  | 0.9 (0.71, 1.15) | 4.04E-01 |
|  | Executive Function Score | 0.88 (0.7, 1.1) | 2.58E-01 |  | 0.9 (0.72, 1.13) | 3.71E-01 |
|  | Hippocampal Volume | 0.9 (0.72, 1.12) | 3.51E-01 |  | 0.92 (0.73, 1.15) | 4.63E-01 |
|  | Entorhinal Cortex Thickness | 1.03 (0.83, 1.28) | 7.72E-01 |  | 1.05 (0.85, 1.3) | 6.54E-01 |
| Oli-M45 | Global Amyloid | **2.99 (2.27, 4.03)** | **6.16E-14** |  | 0.77 (0.4, 1.44) | 4.22E-01 |
|  | Temporal FDG | **0.66 (0.5, 0.85)** | **2.17E-03** |  | 2.39 (1.16, 5.14) | 2.03E-02 |
|  | Memory Score | **0.55 (0.42, 0.71)** | **5.37E-06** |  | 1.67 (0.87, 3.28) | 1.31E-01 |
|  | Executive Function Score | **0.63 (0.49, 0.8)** | **2.14E-04** |  | 0.81 (0.46, 1.46) | 4.64E-01 |
|  | Hippocampal Volume | 0.85 (0.68, 1.05) | 1.35E-01 |  | 1.31 (0.74, 2.35) | 3.55E-01 |
|  | Entorhinal Cortex Thickness | 0.89 (0.71, 1.11) | 3.16E-01 |  | 1.47 (0.8, 2.75) | 2.17E-01 |
| Oli-M50 | Global Amyloid | 1.11 (0.9, 1.37) | 3.38E-01 |  | 1.13 (0.89, 1.43) | 3.10E-01 |
|  | Temporal FDG | 0.9 (0.69, 1.17) | 4.36E-01 |  | 0.89 (0.68, 1.16) | 4.00E-01 |
|  | Memory Score | 1 (0.8, 1.25) | 9.92E-01 |  | 1 (0.79, 1.27) | 9.85E-01 |
|  | Executive Function Score | 1.01 (0.81, 1.27) | 9.26E-01 |  | 1.01 (0.81, 1.28) | 9.07E-01 |
|  | Hippocampal Volume | 1.02 (0.82, 1.26) | 8.82E-01 |  | 1.02 (0.82, 1.26) | 8.77E-01 |
|  | Entorhinal Cortex Thickness | 1.14 (0.92, 1.43) | 2.36E-01 |  | 1.14 (0.92, 1.43) | 2.34E-01 |

**OR (95% CI):** odds ratio with 95% lower and upper bound. Genetic risk-group membership was treated as a binary outcome in the logistic regression model. MRI endophenotypes (hippocampal volume and entorhinal cortex thickness) included intracranial volume and magnetic field strength as covariates. Associations passing multiple testing corrections are bolded P < 2.8x10^-3^.

Supplementary Table 29. Binomial proportion test of differentially expressed genes (DEG) in AD brain cells.

| **Network** | **# of all genes** | **# of prioritized genes** | **% reduction after prioritization** | **Proportion Test** | | |
| --- | --- | --- | --- | --- | --- | --- |
|  |  |  |  | **% of DEGs in prioritized genes** | **% of DEGs in non-prioritized genes** | **P** |
| Ast-M2 | 1032 | 60 | 94% | 78% | 27% | 5.0E-17 |
| Ast-M10 | 449 | 10 | 98% | 40% | 12% | 2.9E-02 |
| Oli-M45 | 1542 | 115 | 93% | 49% | 17% | 1.3E-15 |
| Oli-M50 | 1963 | 98 | 95% | 85% | 27% | 6.1E-33 |

**No. DEG:** number of genes nominally differentially expressed in AD brain cells. **Prop DEG:** proportion of genes differentially expressed.

Supplementary Table 30. Bulk tissue sample characteristics for autopsied brains in FHS.

| **Risk-Group** | **Risk** | **N** | **female** | **Mean Age (sd)** |
| --- | --- | --- | --- | --- |
| Ast-M2 | Low | 38 | 55.26% | 84.24 (10.64) |
|  | High | 41 | 51.22% | 86.16 (7.95) |
| Ast-M10 | Low | 22 | 54.55% | 88 (7.71) |
|  | High | 43 | 62.79% | 86.06 (7.01) |
| Oli-M45 | Low | 39 | 56.41% | 84.44 (10.56) |
|  | High | 41 | 53.66% | 86.27 (7.28) |
| Oli-M50 | Low | 30 | 53.33% | 86.39 (10.26) |
|  | High | 38 | 57.89% | 85.33 (8.25) |

Supplementary Table 31. Differential expression of C4A and AIF1 with genetic risk status in FHS.

| **Gene** | **Ast-M2** | |  | **Ast-M10** | |  | **Oli-M45** | |  | **Oli-M50** | |
| --- | --- | --- | --- | --- | --- | --- | --- | --- | --- | --- | --- |
|  | **LogFC** | **P** |  | **LogFC** | **P** |  | **LogFC** | **P** |  | **LogFC** | **P** |
| *C4A* | 0.75 | 2.24E-02 |  | -0.11 | 7.52E-01 |  | 0.83 | 1.17E-02 |  | 0.24 | 4.16E-01 |
| *IBA1* | -0.34 | 3.45E-01 |  | -0.10 | 7.97E-01 |  | -0.03 | 9.21E-01 |  | 0.27 | 4.80E-01 |

LogFC was calculated using limma and was adjusted for age at exam, sex, and RIN.

**LogFC**: genetic risk group effect on the log2 gene expression.

**Separate excel files:**

**Supplementary Table 32.** Compounds with significant Enrichr Drug_Perturbations_from_GEO enrichment among Ast-M2 prioritized genes.

**Supplementary Table 33.** Compounds with significant Enrichr Drug_Perturbations_from_GEO enrichment among Oli-M45 prioritized genes.

**Supplementary Table 34.** Compounds with significant Enrichr Drug_Perturbations_from_GEO enrichment among Oli-M50 prioritized genes.

**Supplementary Table 35.** Candidate drugs in clinical trials for AD or dementia.

| **Drug** | **Network** | **Clinical Trial Identifier** | **MOA** | **Indication** | **Modulated prioritized Genes** | **Adjusted P** |
| --- | --- | --- | --- | --- | --- | --- |
| Candesartan | Ast-M2, Oli-M45, Oli-M50 | NCT02646982 | angiotensin receptor antagonist | hypertension\|congestive heart failure | HSPB1;SLC3A2;APOE;CLU | 1.90E-03 |
| Cyclophosphamide | Ast-M2 | NCT00013650 | DNA alkylating agent | Hodgkin's lymphoma\|small lymphocytic lymphoma (SLL)\|breast cancer\|neuroblastoma\|Burkitt's lymphoma\|multiple myeloma\|chronic lymphocytic leukemia (CLL)\|acute myeloid leukemia (AML)\|acute lymphoblastic leukemia (ALL)\|chronic myeloid leukemia (CML)\|true histiocytic lymphoma (THL)\|retinoblastoma\|ovarian cancer | EPHX1;SLC3A2;APOE | 7.27E-03 |
| Estradiol | Ast-M2, Oli-M45, Oli-M50 | NCT04312399, NCT00205179, NCT00066157 | estrogen receptor agonist | contraceptive | RPS19;RPLP1;EPHX1;RPS6;APOE;RAMP1;PLTP | 3.36E-07 |
| Isoflurane | Ast-M2, Oli-M50 | NCT01993836, NCT01772537, NCT01870453, NCT01903421 | inhaled anaesthetic | general anaesthetic | HSPB1;SLC3A2;APOE;CLU | 1.07E-03 |
| Levetiracetam | Ast-M2, Oli-M45 | NCT04004702, NCT03489044, NCT02002819, NCT01554683, NCT03461861, NCT01044758, NCT03875638 | calcium channel blocker | seizures | SLC3A2;APOE;CLU | 1.62E-02 |
| Pioglitazone | Ast-M2, Oli-M45, Oli-M50 | NCT00982202, NCT01931566, NCT02284906 | insulin sensitizer\|PPAR receptor agonist | diabetes mellitus | RPS19;RPLP1;RPS6;HSPB1;APOE;CLU;RAMP1 | 8.08E-07 |
| Simvastatin | Ast-M2, Oli-M50 | NCT00053599, NCT00486044, NCT01142336, NCT00939822, NCT00303277, NCT01439555, NCT01978327 | HMGCR inhibitor | hypercholesterolemia\|coronary heart disease\|myocardial infarction\|stroke\|hyperlipidemia | PDGFRB;RPS19;APOE | 4.10E-03 |

Supplementary Table 36. Drug responses with mean differences.

| **Experiment and Treatment** | ***C4A*** | |  | ***C4B*** | |  | ***APOE*** | |
| --- | --- | --- | --- | --- | --- | --- | --- | --- |
|  | **Mean Diff (95% CI)** | **P** |  | **Mean Diff (95% CI)** | **P** |  | **Mean Diff (95% CI)** | **P** |
| **Experiment 1: β-Estradiol** |  |  |  |  |  |  |  |  |
| **DMSO: APOE33 vs APOE44** | -0.31 (-0.55, -0.06) | 0.02 |  | -0.60 (-0.85, -0.35) | <0.0001 |  | -0.33 (-0.60, -0.06) | 0.0178 |
| **Treated: APOE33 vs APOE44** | -0.29 (-0.53, -0.04) | 0.02 |  | -0.27 (-0.51, -0.02) | 0.0329 |  | -0.47 (-0.73, -0.20) | 0.0009 |
| **Treated vs DMSO: APOEE33** | 0.32 (0.08, 0.57) | 0.01 |  | 0.43 (0.18, 0.67) | 0.001 |  | 0.56 (0.30, 0.82) | 0.0001 |
| **Treated vs DMSO: APOEE44** | 0.35 (0.10, 0.59) | 0.01 |  | 0.76 (0.51, 1.01) | <0.0001 |  | 0.42 (0.15, 0.69) | 0.0029 |
| **Experiment 2: Levetiracetam** |  |  |  |  |  |  |  |  |
| **DMSO: APOE33 vs APOE44** | -0.31 (-0.58, -0.04) | 0.0262 |  | -0.60 (-0.84, -0.35) | <0.0001 |  | -0.33 (-0.51, -0.14) | 0.0008 |
| **Treated: APOE33 vs APOE44** | -0.62 (-0.88, -0.36) | <0.0001 |  | -0.51 (-0.75, -0.27) | <0.0001 |  | -0.51 (-0.70, -0.33) | <0.0001 |
| **Treated vs DMSO: APOEE33** | 0.61 (0.35, 0.87) | <0.0001 |  | 0.60 (0.36, 0.84) | <0.0001 |  | 0.65 (0.47, 0.83) | <0.0001 |
| **Treated vs DMSO: APOEE44** | 0.30 (0.03, 0.57) | 0.0302 |  | 0.69 (0.44, 0.93) | <0.0001 |  | 0.47 (0.28, 0.65) | <0.0001 |
| **Experiment 3: Pioglitazone** |  |  |  |  |  |  |  |  |
| **DMSO: APOE33 vs APOE44** | -0.31 (-0.56, -0.05) | 0.0182 |  | -0.50 (-0.76, -0.23) | 0.0005 |  | -0.33 (-0.51, -0.14) | 0.0008 |
| **Treated: APOE33 vs APOE44** | -0.53 (-0.78, -0.28) | <0.0001 |  | -0.54 (-0.81, -0.27) | 0.0002 |  | -0.07 (-0.25, 0.12) | 0.4511 |
| **Treated vs DMSO: APOEE33** | 0.25 (0.01, 0.50) | 0.0434 |  | 0.48 (0.22, 0.74) | 0.0006 |  | 0.55 (0.37, 0.73) | <0.0001 |
| **Treated vs DMSO: APOEE44** | 0.03 (-0.22, 0.28) | 0.801 |  | 0.43 (0.16, 0.71) | 0.0024 |  | 0.81 (0.62, 0.10) | <0.0001 |
| **Experiment 4: Candesartan** |  |  |  |  |  |  |  |  |
| **DMSO: APOE33 vs APOE44** | -0.31 (-0.63, 0.02) | 0.0648 |  | -0.60 (-0.98, -0.22) | 0.0028 |  | -0.33 (-0.65, -0.01) | 0.0409 |
| **Treated: APOE33 vs APOE44** | -0.44 (-0.75, -0.12) | 0.0087 |  | -0.58 (-0.96, -0.20) | 0.0034 |  | -0.50 (-0.81, -0.19) | 0.0021 |
| **Treated vs DMSO: APOEE33** | 0.11 (-0.21, 0.43) | 0.49 |  | 0.01 (-0.36, 0.38) | 0.9681 |  | 0.19 (-0.12, 0.49) | 0.2308 |
| **Treated vs DMSO: APOEE44** | -0.02 (-0.34, 0.31) | 0.9107 |  | 0.02 (-0.36, 0.41) | 0.9094 |  | 0.02 (-0.30, 0.33) | 0.9199 |

Mean Diff: mean difference of E33 minus E44 or untreated minus treated; CI: confidence interval

Supplementary Figure 1. Feature plots of WGCNA networks for Ast and Oli. Modules with a z-summary statistic ≥ 10 were considered preserved. Cell-type specific Feature plots of ROSMAP snRNA data. a. 1^st^ and 2^nd^ TSNE components of RNA expression, colored by cell types. b. Astrocyte feature plots of module eigengene-based connectivity for Ast-M2 and Ast-M10 and *GFAP* expression. c. Oligodendrocyte feature plots of module eigengene-based connectivity for Oli-M45 and Oli-50 and *OLIG1* and *OLIG2* expression.

Supplementary Figure 2. Distribution of quantitative cbPRSs. Relative frequency density of cbPRS scores stratified by cohort.

Supplementary Figure 3. KEGG pathways enriched with priority genes.

Supplementary Figure 4. Expression profiles of PageRank-prioritized genes for Oli-M45 perturbed by top-ranked drugs. These profiles displayed cell-specific expression with distinct biological pathways associated with the priority genes. Stars indicate significance level *p≤0.05, **p≤0.01, ***p≤0.001.

Supplementary Figure 5. Measures of cytotoxicity in *APOE*33 and *APOE*44 human iPSC-derived astrocytes. No cell death or morphological change is observed in any condition.

**Supplementary Figure 6.** Reference genome stability for qPCR normalization.

**Supplementary Figure 7.** Summary of relative expression levels of *C4A*, *C4B,* and *APOE* across biological replicates in a heatmap. The clustering indicates similar expression patterns across samples.

**Supplementary Figure 8.** Measures of drug treatment efficacy in lowering *C4A* and *C4B* expression in *APOE33* and *APOE44* human iPSC-derived astrocytes. Relative expression of *C4A*, *C4B*, and *APOE* genes in *APOE33* (E33) and *APOE44* (E44) iPSC-derived astrocytes treated with candesartan. Gene expression was evaluated by RT-qPCR and presented as relative fold change over the DMSO vehicle in *APOE33* astrocytes. Error bar represented the standard error of the mean. Statistics: n= pooling of 3 iPSC lines per genotype, , 4 biological replicates (wells) and 3 technical replicates from a total of 2 independent differentiation experiments; two-way ANOVA, *p≤0.05, **p≤0.01, ***p≤0.001, ****p≤0.0001.
